# Comparison of Kalman Filters for Inertial Integrated Navigation

**DOI:** 10.3390/s19061426

**Published:** 2019-03-22

**Authors:** Mengde Zhang, Kailong Li, Baiqing Hu, Chunjian Meng

**Affiliations:** Department of Navigation Engineering, Naval University of Engineering, Wuhan 430000, China; 13805328973@163.com (M.Z.); hubaiqing2005@163.com (B.H.); 17771808742@163.com (C.M.)

**Keywords:** integrated navigation, attitude, multiplicative quaternion, extended kalman filter, unscented kalman filter

## Abstract

The current research on integrated navigation is mainly focused on filtering or integrated navigation equipment. Studies systematically comparing and analyzing how to choose appropriate integrated filtering methods under different circumstances are lacking. This paper focuses on integrated navigation filters that are used by different filters and attitude parameters for inertial integrated navigation. We researched integrated navigation filters, established algorithms, and examined the relative merits for practical integrated navigation. Some suggestions for the use of filtering algorithms are provided. We completed simulations and car-mounted experiments for low-cost strapdown inertial navigation system (SINS) to assess the performance of the integrated navigation filtering algorithms.

## 1. Introduction

An integrated navigation system is a system using two or more nonsimilar navigation systems to measure navigation information and calculate or correct the errors of the navigation system from measurements [1,2,3,4]. The integrated navigation system most commonly used is the inertial integrated navigation system because the navigation information is comprehensive and autonomous [5,6,7]. The main part of an inertial integrated navigation system is the inertial navigation system (INS). The INS is mainly divided into two types: platform inertial navigation system (PINS) and strapdown inertial navigation system (SINS). The PINS uses electromechanical control equipment to establish a physical platform, which places low requirements on the navigation computer. Its main disadvantages include its relatively complex structure, huge equipment volume, and heavy weight [8]. With the maturity of optical gyroscopic technology and the development of computers, SINS is gradually replacing PINS, becoming a more popular research topic [9]. SINS replaces the physical platform with a digital platform, which is simpler, smaller, and lighter. However, both PINS and SINS have disadvantages: the navigation accuracy diverges with time due to device errors and self-error-correction is unreliable [10,11,12]. As such, INS needs other navigation systems to form an integrated navigation system.

The inertial integrated navigation system has been used in many fields, such as land navigation, underwater navigation, and even aerospace and unmanned system navigation [13,14,15,16,17,18]. For example, these systems are usually integrated with a global positioning system (GPS), Roland C or an odometer for land navigation integration, and integrated with the Doppler Velocity Log (DVL) baseline system for underwater navigation integration. However, the technological key to achieving inertial integrated navigation is integrated filtering [19].

Filtering is an estimation method used for processing random data, and has been studied and applied as an estimator in related fields. Modern filtering techniques are represented by the Wiener filter and the Kalman filter [20]. The Wiener filter is a frequency domain filter, whereas the Kalman filter is designed in the time domain. The Wiener filter laid the foundation for modern control theory, but the filter experiences problems with real-time calculation and filtering design. Based on the least squares theory, Kalman designed a linear recursive Kalman filter (KF) algorithm. The KF algorithm is simple in structure and easy to execute and calculate on a computer. Although the KF is optimal in the estimation of linear systems, it cannot be applied to nonlinear systems. Farrell, Barth, and other scholars proposed the extended Kalman filter (EKF). It linearizes nonlinear system models using Taylor series and multivariate Jacobian matrices [21]. The EKF and its improved versions are the most widely used nonlinear Kalman filter algorithms in engineering. However, EKF has its drawbacks. If the degree of nonlinearity of the system is high, the estimate accuracy of EKF seriously decreases. In 1995, Julier and Uhlmann proposed the unscented Kalman filter (UKF) algorithm, which does not ignore the high-order terms of Taylor expansion by unscented transform (UT), and thus has high estimate accuracy in nonlinear systems [22]. Emerging filter algorithms, such as cubature Kalman filter (CKF) [23] and particle filter (PF) [24,25], have been theoretically verified in many fields. Zhou J. [24] and Jiao Z. [25] provided methods to reduce the computation complexity of the PF. The research on PF is still in the theoretical stage. Because of its huge calculation, PF is rarely used in integrated navigation filtering methods in engineering practice.

We can apply linear or nonlinear Kalman filters in inertial integrated navigation. These filters are called integrated navigation filters. However, this filtering application is not straightforward because integrated navigation filters have their own particularities in terms of attitude representations. Among the integrated navigation filters, the attitude representations vary. The common representations are rotation angles, the Euler angle, and the family of Rodrigues parameters, such as modified Rodrigues parameters (MRP), and quaternion [26,27]. The earliest integrated navigation filter was constructed from the Euler angle and inertial error equations, which is called Euler-KF in this paper. Since the inertial error equations are linear, the Euler-KF uses KF for filtering. Thus, the Euler-KF is simple and easy to apply. The application of Euler-KF can be traced back to the Apollo moon landing program. In the field of integrated navigation, the Euler-KF is traditionally called indirect integration due to its integrated structure.

Euler-KF can be used in many fields; however, it faces problems with model accuracy due to inertial error equations. To overcome the model accuracy problem with inertial error equations, inertial basic equations have been used in inertial integrated system models with the development of nonlinear filters. Compared with inertial error equations, the inertial basic equations are precision lossless models based on physical law. For inertial basic equations, the key point is how to represent the attitude transformation matrix and nonlinear filtering. The attitude transformation matrix describes a rotation of coordinates using attitude representations. It is the core of the SINS digital platform. For the attitude transformation matrix, different attitude representations can be used. Compared with three-dimensional (3D) attitude parameters like the Euler angle and the family of Rodrigues parameters, the constrained quaternion, as a four-dimensional attitude parameter, is more popular due to global nonsingularity. Then, the quaternion calculation rules can be divided into multiplicative quaternion and additive quaternion. Compared with additive quaternion, the multiplicative quaternion is more widely used because it is more consistent with the definition of quaternion. As the attitude transformation matrix uses quaternion, the filters need to use nonlinear Kalman filters such as EKF and UKF. Inertial integrated navigation based on EKF using multiplicative quaternion is called the multiplicative EKF (MEKF) algorithm.

L.J. Zhang [28] proposed a spacecraft attitude determination algorithm based on MEKF, and I.A. Ruicai et al. [29] applied the MEKF algorithm to low-precision microelectromechanical systems (MEMS) attitude estimation. Fangjun Qin et al. [30] proposed a sequential MEKF that updates the state covariance at each step of the sequential procedure. The essence of MEKF is EKF, so it inherits the disadvantages of EKF. In 2003, Crassidis first proposed a quaternion-based UKF for spacecraft attitude estimation, known as unscented quaternion estimator (USQUE) [31]. The USQUE is a layered filtering algorithm using a nonconstrained rotation vector that represents the attitude error to perform inner layer filtering recursion. In 2006, Crassidis introduced USQUE to land integrated navigation [32]. Zhou J., Edwan E., et al. [33] studied the application of USQUE for tightly integrated MEMS and GPS navigation system. Li Kailong et al. [34] modified USQUE to reduce the switch frequency between error-MRP and quaternion.

For attitude transformation matrixes, quaternion is not the only choice. Euler angles and the family of Rodrigues parameters can be also used for attitude transformation matrix. However, they have an attitude singularity problem. MRP can use its shadow MRP (SMRP) to avoid singularity. Karlgaard and Schaub proposed MRP-EKF and MRP-UKF using MRP and SMRP for inertial integrated navigation [35]. Recently, some studies focused on Euler angles for nonlinear inertial integrated filtering. With Euler angles, different rotation orders can form different Euler angles. Different Euler angles have different singularities. Thus, Euler angles can use a special rotation order to avoid singularity. Ran and Cheng [36] used double Euler angles under UKF for initial alignment, which is called DoEuler-UKF in this paper. Although still some problems exist with DoEuler-UKF, DoEuler-UKF has special advantages, like clear physical meaning and simpler algorithm structure. We think the DoEuler-UKF will be extensively researched.

In this paper, we focus on Euler-KF, MEKF, USQUE, MRP-UKF, and DoEuler-UKF. These five filters are currently common or research hotspots in integrated filtering methods. Although some studies proposed those five filters, systematic research comparing these filters in inertial integrated navigation is lacking. The published research on Kalman filters in the navigation field mainly focus on attitude estimation and filter improvement. To fill the literature gap about the characteristics and applicable settings of low-precision inertial navigation integrated navigation, this paper focuses on those filters, especially in terms of algorithm establishment, characteristics, the merits of the specific integrated navigation system, and the applicable environment. Through research and comparison of these five filters using simulation tests and a loosely coupled MEMS/GPS experiment, the performance of these filtering algorithms was systematically analyzed. As a result, we drew some conclusions about the various indicators of the five filters. Some suggestions are as a basis for the selection of integrated filtering methods in different situations.

The organization of this paper is as follows. In the review of existing theories, the basic SINS equations and the SINS error equations are introduced. In the filtering algorithm section, MEKF and USQUE are studied in detail. Finally, simulation testing and car-mounted experiments were conducted and are summarized in the experimental section. The results are provided and the performance of the five inertial integrated navigation filters are compared. A few meaningful conclusions are outlined in the summary.

## 2. Theoretical Review

In this section, the reference frames commonly used to derive the strapdown inertial integrated navigation system, the properties of basic SINS equations, and the error SINS equations are provided.

The coordinate frame is the research foundation of SINS. The SINS attitude transformation matrix is usually related to two important coordinate frames: body frame (b-frame, Right-Front-Up, RFU) and navigation frame (n-frame, East-North-Up, ENU). The b-frame is fixed onto the vehicle body and rotates with it. The outputs of IMU are expressed with respect to the b-frame. The n-frame is the reference frame that performs navigation calculation. Other coordinates used in this paper are the Earthframe (e-frame) and the inertial frame (i-frame). The outputs of IMU of SINS should be transformed from b-frame to n-frame for navigation calculation using the attitude transformation matrix. The vector ***r**^b^* with respect to b-frame can be transformed to ***r**^n^* with respect to n-frame:

(1) $$r^{n}=C_{b}^{n}r^{b}$$

(2) $$C_{b}^{n}={\left(C_{n}^{b}\right)}^{T}$$

where $C_{b}^{n}$ is the attitude transformation matrix used to describe a rotation of coordinates using different attitude representations.

### 2.1. Basic SINS Equations

Basic SINS equations are the core of the system model of integrated filtering methods, which contain an attitude part, a velocity part, a position part, a gyro measurement part, and an accelerometer measurement part. For basic SINS equations, the key parts are the attitude transformation matrix and attitude kinematic equation because they form the essence of the SINS digital platform. The attitude transformation can be represented by the Euler angle, MRP, and quaternion. When using the Euler angle, the attitude kinematic equation is given by

(3) $$\left[\begin{array}{c} \dot{\theta }\\ \dot{\gamma }\\ \dot{\psi } \end{array}\right]=\frac{1}{\cos\theta }\left[\begin{array}{ccc} \cos\theta \cos\gamma & 0 & \cos\theta \sin\gamma \\ \sin\theta \sin\gamma & \cos\theta & -\cos\gamma \sin\theta \\ -\sin\gamma & 0 & \cos\gamma \end{array}\right]\omega_{nb}^{b}$$

where $\varphi =\left[\theta ;\gamma ;\psi \right]$, and θ is pitch, γ is roll, ψ is yaw, $\omega_{nb}^{b}$ is angular velocity of the *b*-frame relative to the *n*-frame, $\omega_{ib}^{b}$ is the gyro output, and $\omega_{in}^{n}$ is the angular velocity of the *n*-frame relative to the *i*-frame. The attitude transformation matrix $C_{n}^{b}(\varphi )$ using the Euler angle is given by

(4) $$C_{n}^{b}(\varphi )=\left[\begin{array}{ccc} \cos\gamma \cos\psi +\sin\gamma \sin\psi \sin\theta & \sin\psi \cos\theta & \sin\gamma \cos\psi -\cos\gamma \sin\psi \sin\theta \\ -\cos\gamma \sin\psi +\sin\gamma \cos\psi \sin\theta & \cos\psi \cos\theta & -\sin\gamma \sin\psi -\cos\gamma \cos\psi \sin\theta \\ -\sin\gamma \cos\theta & \sin\theta & \cos\gamma \cos\theta \end{array}\right]$$

When using MRP, the attitude kinematic equation is

(5) $$\dot{\sigma }=\frac{1}{4}\left\{\left(1-{\left\|\sigma \right\|}^{2}\right)I_{3\times 3}+2\left[\sigma \times \right]+2\sigma \sigma^{T}\right\}\omega_{nb}^{b}$$

where σ is MRP and $I_{3\times 3}$ is a unit matrix. The attitude transformation matrix $C_{n}^{b}(\sigma )$ using MRPis given by

(6) $$C_{n}^{b}(\sigma )={\left(\frac{I_{3\times 3}-\left[\sigma \times \right]}{I_{3\times 3}+\left[\sigma \times \right]}\right)}^{2}=I_{3}+\frac{8{\left[\sigma \times \right]}^{2}-4\left(1-{\left\|\sigma \right\|}^{2}\right)\left[\sigma \times \right]}{{\left(1+{\left\|\sigma \right\|}^{2}\right)}^{2}}$$

Then, the attitude kinematic equation is:

(7) $$\dot{q}=\frac{1}{2}\Xi (q)\omega_{nb}^{b}=\frac{1}{2}\Omega (\omega_{nb}^{b})q$$

where $q=\left[\rho ;q_{4}\right]$ is a quaternion with $\rho =\left[q_{1};q_{2};q_{3}\right]$ vector part and $q_{4}$ scalar part and $\Omega (\omega_{nb}^{b})=\left[\begin{array}{cc} -[\omega_{nb}^{b}\times ] & \omega_{nb}^{b}\\ -{\left(\omega_{nb}^{b}\right)}^{T} & 0 \end{array}\right]$. Then, the $\left(\cdot \times \right)$ cross product matrix is defined by

$$\left[x\times \right]=\left[\begin{array}{ccc} 0 & -x_{3} & x_{2}\\ x_{3} & 0 & x_{1}\\ -x_{2} & x_{1} & 0 \end{array}\right]\;\mathrm{with}\;x=\left[x_{1};x_{2};x_{3}\right]$$

The attitude transformation matrix $C_{b}^{n}(q)$ using a quaternion is

(8) $$\begin{array}{l} C_{n}^{b}(q)=\Psi {\left(q\right)}^{T}\Xi (q)=\left[\begin{array}{ccc} q_{1}^{2}-q_{2}^{2}-q_{3}^{2}+q_{4}^{2} & 2(q_{1}q_{2}-q_{3}q_{4}) & 2(q_{1}q_{3}+q_{2}q_{4})\\ 2(q_{1}q_{2}+q_{3}q_{4}) & -q_{1}^{2}+q_{2}^{2}-q_{3}^{2}+q_{4}^{2} & 2(q_{2}q_{3}-q_{1}q_{4})\\ 2(q_{1}q_{3}-q_{2}q_{4}) & 2(q_{2}q_{3}+q_{1}q_{4}) & -q_{1}^{2}-q_{2}^{2}+q_{3}^{2}+q_{4}^{2} \end{array}\right] \end{array}$$

where

$$\Xi (q)=\left[\begin{array}{l} q_{4}I_{3\times 3}+\left[\rho \times \right]\\ \begin{array}{ccc} & -\rho^{T} & \end{array} \end{array}\right]\;\mathrm{and}\;\Psi (q)=\left[\begin{array}{l} q_{4}I_{3\times 3}-\left[\rho \times \right]\\ \begin{array}{ccc} & -\rho^{T} & \end{array} \end{array}\right]$$

where ***I***_3×3_ is a 3 × 3 unit matrix. The attitude kinematic equation is different using different attitude representations. This is the core of basic SINS equations. Then, other parts of the basic SINS equations are [37]

(9) $$\dot{\lambda }=\frac{v_{N}}{R_{M}+h}$$

(10) $$\dot{\phi }=\frac{v_{E}}{(R_{N}+h)\cos\lambda }$$

(11) $$\dot{h}=v_{U}$$

(12) $${\dot{v}}_{E}=\left[\frac{v_{E}}{(R_{N}+h)\cos\lambda }+2\omega_{ie}^{e}\right]v_{N}\sin\lambda -\frac{v_{E}v_{U}}{R_{N}+h}-2\omega_{ie}^{e}v_{U}\cos\lambda +f_{E}$$

(13) $${\dot{v}}_{N}=-\left[\frac{v_{E}}{(R_{N}+h)\cos\lambda }+2\omega_{ie}^{e}\right]v_{E}\sin\lambda -\frac{v_{E}v_{U}}{R_{M}+h}+f_{N}$$

(14) $${\dot{v}}_{U}=\frac{v_{E}^{2}}{R_{N}+h}+\frac{v_{N}^{2}}{R_{M}+h}+2\omega_{ie}^{e}v_{E}\cos\lambda -g-f_{U}$$

where (9)–(11)are position kinematic equations and (12)–(14) are velocity kinematic equations; is the position, λ is the latitude, ϕ is the longitude, and h is the height; $v=\left[v_{E}^{};v_{N}^{};v_{U}^{}\right]$ is the velocity; g is the gravity vector; is the Earth’s rotation rate, which is 7.292115 × 10^−5^ rad/s (WGS-84); is the specific force vector in *n*-frame, expressed in the *b*-frame by; $\omega_{ib}^{b}$ is the gyro measurements; and $R_{M}$ and $R_{M}$ are the radius of Earth:

(15) $$R_{M}={R_{e}(1-e^{2})/\left(1-e^{2}\sin^{2}\lambda \right)}^{1.5}$$

(16) $$R_{N}={R_{e}/\left(1-e^{2}\sin^{2}\lambda \right)}^{0.5}$$

where $R_{e}$= 6,378,137 m and e = 0.0818. The gyro measurement equation is

(17) $${\tilde{\omega }}_{ib}^{b}=\omega_{ib}^{b}+\varepsilon +\eta_{gv}$$

(18) $$\dot{\varepsilon }=\eta_{gu}$$

where ${\tilde{\omega }}_{ib}^{b}$ is the outputs of the gyro with the gyro bias; $\eta_{gv}$ and $\eta_{gu}$ are zero-mean Gaussian white-noise process with spectral densities given by $\sigma_{gv}^{2}$ and $\sigma_{gu}^{2}$, respectively. The accelerometer measurement equation is

(19) $${\tilde{f}}^{b}=f^{b}+\nabla +\eta_{av}$$

(20) $$\dot{\nabla }=\eta_{au}$$

where ${\tilde{f}}^{b}$ is the outputs of the accelerometer with accelerometer bias ∇, and $\eta_{av}$ and $\eta_{au}$ are zero-mean Gaussian white-noise process with spectral densities given by $\sigma_{av}^{2}$ and $\sigma_{au}^{2}$, respectively.

### 2.2. SINS Error Equations

The SINS error equations can be derived from the basic SINS equations. As with basic SINS equations, SINS error equations have an attitude error part, velocity error part, and position error part. The key process derived from basic SINS equations is how to present the attitude error equation. The attitude error equation reflects the transition from quaternion to Euler angle error δφ. The velocity error equation and position error equation are mainly formed by assuming the approximation condition, simplification, and ignoring the infinitesimal term. As such, the SINS error equations can be obtained. By analyzing the derivation principle of the SINS error equations, the SINS error equations affect the integrated navigation estimate effect to a large extent in some cases when the motion is severe or under large misalignment angles. For SINS error equations, the importance is the attitude equations using the Euler angle error. The attitude error equations are

(21) $$\delta {\dot{\varphi }}_{E}=\delta \varphi_{N}\left(\omega_{ie}^{e}\sin\lambda +\frac{v_{E}\tan\lambda }{R_{N}+h}\right)-\delta \varphi_{U}\left(\omega_{ie}^{e}\cos\lambda +\frac{v_{E}}{R_{N}+h}\right)-\frac{\delta v_{N}}{R_{M}+h}+\delta h\frac{v_{N}}{{\left(R_{M}+h\right)}^{2}}-\varepsilon_{E}^{b}$$

(22) $$\delta {\dot{\varphi }}_{N}=-\delta \varphi_{E}\left(\omega_{ie}^{e}\sin\lambda +\frac{v_{E}\tan\lambda }{R_{N}+h}\right)-\delta \varphi_{U}\frac{v_{N}}{R_{M}+h}-\delta \lambda \omega_{ie}^{e}\sin\lambda +\frac{\delta v_{E}}{R_{N}+h}-\delta h\frac{v_{E}}{{\left(R_{N}+h\right)}^{2}}-\varepsilon_{N}^{b}$$

(23) $$\begin{array}{l} \delta {\dot{\varphi }}_{U}=-\delta \varphi_{E}\left(\omega_{ie}^{e}\cos\lambda +\frac{v_{E}}{R_{N}+h}\right)+\frac{\delta \varphi_{N}v_{N}}{R_{M}+h}+\delta \lambda \left(\omega_{ie}^{e}\cos\lambda +\frac{v_{E}}{R_{N}+h}\sec^{2}\lambda \right)\\ \;+\frac{\delta v_{E}\tan\lambda }{R_{N}+h}-\delta h\frac{v_{E}\tan\lambda }{{\left(R_{N}+h\right)}^{2}}-\varepsilon_{U}^{b} \end{array}$$

where δ(⋅) is the position or velocity error. The other velocity or position parts can be obtained from corresponding references. Then, the SINS error equations are linear and the KF will be optimal for solving the filtering problem. Four aspects of connections and differences between basic SINS equations and error equations are summarized as follows:

(1) The SINS error equations are formed by the equivalent transformation and approximation processing of the basic SINS equations
(2) The SINS error equations are linear equations, and the basic SINS equations are nonlinear equations.
(3) Compared with the SINS error equations, the basic SINS equations directly reflect the change in navigation parameters, and therefore can more accurately reflect the actual motion of the carrier.
(4) For the attitude error equation, the linearization premise is that the attitude error angle is a small angle, so for large misalignment angles, the equation loses the linear assumption and becomes a nonlinear equation.

## 3. Kalman Filters for Inertial Integrated Navigation

**Figure 1:**
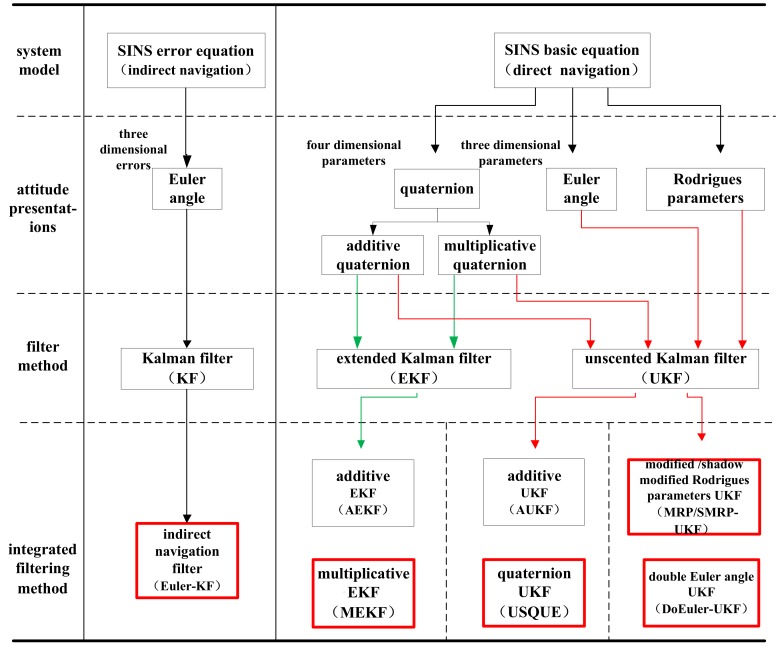
The relationship between attitude, model, and filtering methods.

For SINS, the first step is to determine the attitude representation. The second step involves selecting the corresponding Kalman filter according to the integrated navigation system model. The relationships between attitude representation, model, and integrated filtering methods are shown in Figure 1.

For the SINS error equations, the Euler-KF is used for estimation. For the basic SINS equations, nonlinear Kalman filters can be used for estimation. Then, the nonlinear Kalman filters, like EKF and UKF with different attitude representations, can form different integrated navigation filters. EKF includes additive EKF (AEKF) and MEKF using quaternion. Compared with MEKF, AEKF is rarely used in engineering. Thus, we mainly studied MEKF. UKF includes additive UKF (AUKF) and USQUE using a quaternion. Although AUKF may exist in theory, we were unable to find any published literature on this filter. Thus, we mainly studied USQUE. Finally, some 3Dattitude representations, like MRP and Euler angle, were used in UKF for integrated navigation filters: MRP-UKF and DoEuler-UKF. For the 3D integrated navigation filters, the main problem is how to avoid singularity. Thus, we discussed the singularity avoidance of MRP-UKF and DoEuler-UKF.

### 3.1. Multiplicative Extended Kalman Filter

The attitude quaternion parameter normalization constraint is easily satisfied for the calculation of a single quaternion. However, in the EKF nonlinear filter calculation, problems may be encountered. In addition, the covariance matrix in EKF is 3 × 3, whereas a quaternion is a four-dimensional parameter, thus EKF cannot be directly applied to the strapdown inertial integrated navigation system with a quaternion due to the dimension mismatch. The basic idea of MEKF is to reduce the dimension by a small error quaternion. The unconstrained 3D attitude error parameters were estimated using multiplicative quaternions to provide global nonsingular attitude descriptions. For the nonlinear model using nonlinear function local linear features, we applied the first-order Taylor expansion to the model, and obtained the Jacobian matrix of the model, so the Jacobian matrix could be applied to the propagation of the covariance matrix. Since the difference between the MEKF algorithm and the EKF algorithm is caused by attitude, we emphasized the attitude part of the algorithm. The attitude part mainly influences the propagation of covariance.

The quaternion has no physical meaning. The MEKF algorithm reduces the dimensionality of the error quaternion by approximating the quaternion attitude and the real quaternion attitude δq, which is reduced to the three-dimensional δα, so that the Jacobian matrix can be obtained. The covariance matrix can be propagated. In the measurement update, due to the quaternion attitude, the quaternion attitude cannot be expressed in a unified form with the position, velocity, or other information about the 3D vector. Therefore, the measurement update of the attitude is expressed by the quaternion. After the measurement update, the state update is added to complete the estimation of the states.

In the following part, we derive the MEKF process combined with the strapdown inertial integrated navigation system. The state chooses quaternion attitude, position, velocity, gyro bias, and accelerometer bias. Due to the dimension problem, the state vectors are divided into an attitude part and an ‘other’ part, so we define the state ${\hat{x}}_{k-1}^{}={\left[{\hat{x}}_{k-1}^{q},{\hat{x}}_{k-1}^{e}\right]}^{T}$ and covariance $P_{k-1}$. ${\hat{x}}_{k-1}^{q}$ is quaternion attitude and ${\hat{x}}_{k-1}^{e}$ is the other states ${\hat{x}}_{k-1}^{e}={\left[{\hat{p}}_{k-1},{\hat{v}}_{k-1}^{},{\hat{\varepsilon }}_{k-1}^{},{\hat{\nabla }}_{k-1}^{}\right]}^{T}$. The discrete time state inertial integrated navigation process model can be given as $x_{k}=f(x_{k-1})+w_{k-1}$.

The basic goal of MEKF is to reduce the dimension with an error quaternion. MEKF uses the error quaternion as the state vector in the time update covariance propagation using the multiplicative rule. MEKF starts by the definition of the error quaternion. The error quaternion is defined by the multiplicative error as

(24) $$\delta q=q⊗{\hat{q}}^{-1}\;\mathrm{with}\;\delta q=\left[\delta \rho ;\delta q_{4}\right].$$

The attitude error matrix is given by

(25) $$C_{b}^{n}(\delta q)=C_{b}^{n}(q){\left[C_{b}^{n}(\hat{q})\right]}^{T}.$$

For small angles the vector part of the quaternion is approximately equal to half angles, then δρ≈δα/2 and $\delta q_{4}\approx 1$, where δα is a small error-angle rotation vector. The linearized model error-kinematics is as follows:

(26) $$\delta \dot{\alpha }=-[{\hat{\omega }}_{nb}^{b}\times ]\delta \alpha +\delta \omega_{ib}^{b}-C_{b}^{n}(\hat{q})\delta \omega_{in}^{n}$$

(27) $$\delta {\dot{q}}_{4}=0$$

where $\delta \omega_{ib}^{b}=\omega_{ib}^{b}-{\hat{\omega }}_{ib}^{b}$ and $\delta \omega_{in}^{n}=\omega_{in}^{n}-{\hat{\omega }}_{in}^{n}$. $\delta \omega_{in}^{n}$ can be computed by first-order Taylor series expansion. Usually, the true quaternion is close to the estimated quaternion. Therefore, we can reduce the quaternion to a three-dimensional vector:

(28) $$\delta \omega_{ib}^{b}=-[I_{3\times 3}\Delta \varepsilon +I_{3\times 3}\eta_{gv}]\;\mathrm{with}\;\Delta \varepsilon =\varepsilon -\hat{\varepsilon }$$

Then,

(29) $$\delta \dot{\alpha }=-\left[({\tilde{\omega }}_{ib}^{b}-\hat{\varepsilon })\times \right]\delta \alpha -\Delta \varepsilon -\eta_{gv}-C_{n}^{b}{\left.(q)\frac{\partial \omega_{in}^{n}}{\partial p}\right|}_{\hat{p},\hat{v}}\Delta p-C_{n}^{b}{\left.(q)\frac{\partial \omega_{in}^{n}}{\partial v}\right|}_{\hat{p}}\Delta v$$

where $\Delta p=p-\hat{p}$ and $\Delta v=v-\hat{v}$. The partials are shown as

(30) $$\frac{\partial \omega_{in}^{n}}{\partial p}=\left[\begin{array}{ccc} \frac{v_{N}}{{\left(R_{M}^{}+h\right)}^{2}}\frac{\partial R_{M}}{\partial \lambda } & 0 & \frac{v_{N}}{{\left(R_{M}^{}+h\right)}^{2}}\\ -\omega_{ie}^{e}\sin\lambda -\frac{v_{E}}{{\left(R_{N}^{}+h\right)}^{2}}\frac{\partial R_{N}}{\partial \lambda } & 0 & -\frac{v_{E}}{{\left(R_{N}^{}+h\right)}^{2}}\\ \omega_{ie}^{e}\cos\lambda +\frac{v_{E}\sec^{2}\lambda }{R_{N}^{}+h}-\frac{v_{E}\tan\lambda }{{\left(R_{N}^{}+h\right)}^{2}}\frac{\partial R_{N}}{\partial \lambda } & 0 & -\frac{v_{E}\tan\lambda }{{\left(R_{N}^{}+h\right)}^{2}} \end{array}\right]$$

(31) $$\frac{\partial \omega_{in}^{n}}{\partial v}=\left[\begin{array}{ccc} 0 & -\frac{1}{R_{M}+h} & 0\\ \frac{1}{R_{N}^{}+h} & 0 & 0\\ \frac{\tan\lambda }{R_{N}^{}+h} & 0 & 0 \end{array}\right]$$

where $\frac{\partial R_{N}}{\partial \lambda }=\frac{R_{e}e^{2}\sin\lambda \cos\lambda }{{\left(1-e^{2}\sin n^{2}\lambda \right)}^{3/2}}$ and $\frac{\partial R_{M}}{\partial \lambda }=\frac{3R_{e}(1-e^{2})e^{2}\sin\lambda \cos\lambda }{{\left(1-e^{2}\sin^{2}\lambda \right)}^{5/2}}$. After the dimension reduction is completed, the calculation of the Jacobian matrix can be performed to propagate the covariance matrix. Then, we determine the state error $\Delta x={\left[\delta \alpha \left({\hat{q}}_{k-1}\right),\Delta {\hat{p}}_{k-1},\Delta {\hat{v}}_{k-1}^{},\Delta {\hat{\varepsilon }}_{k-1}^{},\Delta {\hat{\nabla }}_{k-1}^{}\right]}^{T}$. The state, state error, noise vector, and covariance used in MEKF are given by

(32) $$x=\left[q;p;v;\varepsilon ;\nabla \right],\Delta x=\left[\delta \alpha ;\Delta p;\Delta v;\Delta \varepsilon ;\Delta \nabla \right],w=\left[\eta_{gv};\eta_{gu};\eta_{av};\eta_{au}\right]$$

(33) $$Q=\left[\begin{array}{cccc} \sigma_{gv}^{2}I_{3\times 3} & 0_{3\times 3} & 0_{3\times 3} & 0_{3\times 3}\\ 0_{3\times 3} & \sigma_{gu}^{2}I_{3\times 3} & 0_{3\times 3} & 0_{3\times 3}\\ 0_{3\times 3} & 0_{3\times 3} & \sigma_{av}^{2}I_{3\times 3} & 0_{3\times 3}\\ 0_{3\times 3} & 0_{3\times 3} & 0_{3\times 3} & \sigma_{au}^{2}I_{3\times 3} \end{array}\right]$$

where $0_{3\times 3}$ is a 3 × 3 zeros matrix. After propagating filtering state, MEKF propagates covariance using an error equation. Then, the error equation is given by

(34) $$\Delta \dot{x}=F\Delta x+Gw$$

where

(35) $$F=\left[\begin{array}{ccccc} F_{11} & F_{12} & F_{13} & F_{14} & 0_{3\times 3}\\ 0_{3\times 3} & F_{22} & F_{23} & 0_{3\times 3} & 0_{3\times 3}\\ F_{31} & F_{32} & F_{33} & 0_{3\times 3} & F_{35}\\ 0_{3\times 3} & 0_{3\times 3} & 0_{3\times 3} & 0_{3\times 3} & 0_{3\times 3}\\ 0_{3\times 3} & 0_{3\times 3} & 0_{3\times 3} & 0_{3\times 3} & 0_{3\times 3} \end{array}\right].$$

The system noise is $w=\left[\eta_{gv};\eta_{gu};\eta_{av};\eta_{au}\right]$ and covariance is

(36) $$Q=\left[\begin{array}{cccc} \sigma_{gv}^{2}I_{3\times 3} & 0_{3\times 3} & 0_{3\times 3} & 0_{3\times 3}\\ 0_{3\times 3} & \sigma_{gu}^{2}I_{3\times 3} & 0_{3\times 3} & 0_{3\times 3}\\ 0_{3\times 3} & 0_{3\times 3} & \sigma_{av}^{2}I_{3\times 3} & 0_{3\times 3}\\ 0_{3\times 3} & 0_{3\times 3} & 0_{3\times 3} & \sigma_{au}^{2}I_{3\times 3} \end{array}\right]$$

and the noise coefficient is

(37) $$G=\left[\begin{array}{cccc} -I_{3\times 3} & 0_{3\times 3} & 0_{3\times 3} & 0_{3\times 3}\\ 0_{3\times 3} & 0_{3\times 3} & 0_{3\times 3} & 0_{3\times 3}\\ 0_{3\times 3} & 0_{3\times 3} & -C_{b}^{n}(\hat{q}) & 0_{3\times 3}\\ 0_{3\times 3} & I_{3\times 3} & 0_{3\times 3} & 0_{3\times 3}\\ 0_{3\times 3} & 0_{3\times 3} & 0_{3\times 3} & I_{3\times 3} \end{array}\right]$$

with Table 1.

The position partial differentials are

(38) $$\frac{\partial \dot{p}}{\partial p}=\left[\begin{array}{ccc} -\frac{v_{N}}{{\left(R_{M}^{}+h\right)}^{2}}\frac{\partial R_{M}}{\partial \lambda } & 0 & -\frac{v_{N}}{{\left(R_{M}^{}+h\right)}^{2}}\\ -\frac{v_{E}\sec\lambda }{{\left(R_{N}^{}+h\right)}^{2}}\frac{\partial R_{N}}{\partial \lambda }+\frac{v_{E}\sec\lambda \tan\lambda }{R_{N}^{}+h} & 0 & -\frac{v_{E}\sec\lambda }{{\left(R_{N}^{}+h\right)}^{2}}\\ 0 & 0 & 0 \end{array}\right]$$

(39) $$\frac{\partial \dot{p}}{\partial v}=\left[\begin{array}{ccc} 0 & \frac{1}{R_{M}+h} & 0\\ \frac{\sec\lambda }{R_{N}^{}+h} & 0 & 0\\ 0 & 0 & 1 \end{array}\right]$$

The velocity partial differentials are

(40) $$\frac{\partial \dot{v}}{\partial p}=\left[\begin{array}{ccc} Y_{11} & 0 & Y_{13}\\ Y_{21} & 0 & Y_{23}\\ Y_{31} & 0 & Y_{33} \end{array}\right]$$

(41) $$\frac{\partial \dot{v}}{\partial v}=\left[\begin{array}{ccc} Z_{11} & Z_{12} & Z_{13}\\ Z_{21} & Z_{22} & Z_{23}\\ Z_{31} & Z_{32} & 0 \end{array}\right]$$

with Table 2 and Table 3.

$$\frac{\partial g}{\partial \lambda }=9.780327\cdot \left[1.06048\times 10^{-2}\sin\lambda \cos\lambda -4.64\times 10^{-5}(\sin\lambda cos^{3}\lambda -\sin^{3}\lambda \cos\lambda )\right]$$

$$\frac{\partial g}{\partial h}=-3.0877\times 10^{-6}+4.4\times 10^{-9}\sin^{2}\lambda +1.44\times 10^{-13}h$$

In the measurement update, either the method of tightly coupled or super tightly coupled is adopted, then the measurement equation is nonlinear. For the MEKF algorithm, the filtering process of the measurement update is basically the same as the time update. Using the loosely-coupled model, the measurement equation is a linear equation. The difference from Kalman filtering is that, since the quaternion attitude dimension does not match other state quantities, $\delta {\hat{\alpha }}_{k}$ and $\delta {\hat{x}}_{k}^{e}$ are separately expressed in the measurement update. We introduce the measurement update by taking the loosely-coupled model as an example.

(42) $$y_{k}=H_{k}x_{k}+\eta_{k}$$

where $H_{k}$ varies with the change in the measurement. When only using position information as the loosely-coupled measurement transfer matrix,

(43) $$H_{p,k}=[\begin{array}{ccc} 0_{3\times 3} & I_{3\times 3} & 0_{3\times 9} \end{array}]$$

When only using velocity information as the loosely-coupled measurement transfer matrix:

(44) $$H_{v,k}=[\begin{array}{ccc} 0_{3\times 6} & I_{3\times 3} & 0_{3\times 6} \end{array}].$$

When using both position and velocity information as the loosely-coupled measurement transfer matrix:

(45) $$H_{p,v,k}=\left[\begin{array}{cccc} 0_{3\times 3} & I_{3\times 3} & 0_{3\times 3} & 0_{6\times 3}\\ 0_{3\times 3} & 0_{3\times 3} & I_{3\times 3} & 0_{6\times 3} \end{array}\right].$$

In the state update, the entire filtering process is completed by updating the state vectors by using the errors. The MEKF algorithm is summarized in Algorithm 1.

**Algorithm 1:** The multiplicative extended Kalman filter (MEKF) algorithm.*Time Propagation*:Define the filtering state ${\hat{x}}_{k-1}\left(\hat{q}\right)$ and $P_{k-1}$.Propagate filtering state and covariance 
${\hat{x}}_{k/k-1}(\hat{q})=f\left({\hat{x}}_{k-1}(\hat{q})\right)$

$P_{k/k-1}=\Phi_{k-1}P_{k-1}\Phi_{k-1}^{T}+W_{k-1}$
where $\Phi_{k-1}$ is the discrete-time state transition matrix obtained via numerical solution.*Measurement update*:Compute filtering gain $K_{k}=P_{k/k-1}H_{k}^{T}{\left(H_{k}P_{k/k-1}H_{k}^{T}+R_{k}\right)}^{-1}$Compute state error $\delta {\hat{x}}_{k}=K_{k}\left[y_{k}-h({\hat{x}}_{k/k-1})\right]=\left[\begin{array}{c} \delta {\hat{\alpha }}_{k}^{}\\ \delta {\hat{x}}_{k}^{e} \end{array}\right]$ with $\delta {\hat{x}}_{k}^{e}=\left[\delta {\hat{p}}_{k};\delta {\hat{v}}_{k}^{};\delta {\hat{\varepsilon }}_{k}^{};\delta {\hat{\nabla }}_{k}^{}\right]$
Compute quaternion states
${\hat{q}}_{k}={\hat{q}}_{k/k-1}+\frac{1}{2}\Xi ({\hat{q}}_{k/k-1})\delta {\hat{\alpha }}_{k}$
Compute other states
${\hat{x}}_{k}^{e}={\hat{x}}_{k/k-1}^{e}+\delta {\hat{x}}_{k}^{e}$
Compute filtering covariance $P_{k}=(I_{n}-K_{k}H_{k})P_{k/k-1}$


### 3.2. Unscented Quaternion Estimator

**Figure 2:**
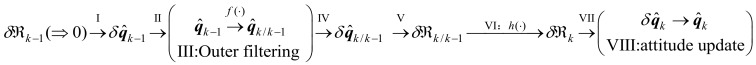
USQUE algorithm.

Because UKF cannot be directly applied to the strapdown inertial integrated navigation system with quaternion presentation attitude, guaranteeing solutions to the attitude quaternion constraint problem of the attitude quaternion parameter and the matching constraint problem of the quaternion filter variance matrix is difficult. USQUE solves the problem of UKF’s attitude estimation in the equation of state is “layered filtering”. The filtering process is divided into two layers: the inner layer filtering uses the generalized error Rodrigues parameter to update the attitude state, and the outer layer filtering uses the quaternion to perform the attitude. The quaternion and the generalized error Rodrigues parameter use the attitude transformation relationship of the multiplicative error quaternion as a bridge for switching. The basic idea of USQUE is shown in Figure 2.

According to Figure 2, the USQUE filtering process can be summarized. The equation of state and the measurement equation have been described in detail above. Similar to MEKF, the state is divided into two parts: attitude vector and nonattitude vector. The nonattitude part is like MEKF but the attitude part is a generalized Rodrigues parameter (GRP) ${\hat{x}}_{k-1}^{\delta ℜ}$, where the state vector is ${\hat{x}}_{k-1}=\left[{\hat{x}}_{k-1}^{\delta ℜ};{\hat{x}}_{k-1}^{e}\right]$. The corresponding filter covariance matrix is $P_{k-1}$. The USQUE algorithm is used to estimate ${\hat{x}}_{k}^{\delta ℜ}$ and then the corresponding quaternion attitude ${\hat{x}}_{k}^{q}$ in the state estimation. For the loose-coupled method, the measurement equation is a linear equation and the measurement update process is the same as for the linear Kalman filter. After the measurement update is completed, the attitude GRP is converted to a quaternion, which is the purpose of the attitude update. The algorithm is summarized in Algorithm 2.

**Algorithm 2:** The unscented quaternion estimator (USQUE) algorithm.*Time Propagation*:Define the filtering state Using error MRP describe attitude ${\hat{x}}_{k-1}^{}(\delta ℜ)=\left[{\hat{x}}_{k-1}^{\delta ℜ};{\hat{x}}_{k-1}^{e}\right]$
Using a quaternion describe attitude ${\hat{x}}_{k-1}^{}(q)=\left[{\hat{x}}_{k-1}^{q};{\hat{x}}_{k-1}^{e}\right]$ and covariance $P_{k-1}$Create sigma points using UT $\chi_{k-1}\left(i\right)=\left[\begin{array}{c} \chi_{k-1}^{\delta ℜ}\left(i\right)\\ \chi_{k-1}^{e}\left(i\right) \end{array}\right]=sigma\left({\hat{x}}_{k-1}\left(\delta ℜ\right),P_{k-1}\right)$
The $\chi_{k-1}^{\delta ℜ}\left(i\right)$ corresponding quaternion error $\chi_{k-1}^{\delta q}\left(i\right)=\left[\delta \rho_{k-1}^{}\left(i\right);\delta q_{4,k-1}\left(i\right)\right]$

$\delta q_{4,k-1}\left(i\right)=\frac{-a{\left\|\chi_{k-1}^{\delta ℜ}\left(i\right)\right\|}^{2}+f\sqrt{f^{2}+\left(1-a^{2}\right){\left\|\chi_{k-1}^{\delta ℜ}\left(i\right)\right\|}^{2}}}{f^{2}+{\left\|\chi_{k-1}^{\delta ℜ}\left(i\right)\right\|}^{2}}$

$\delta \rho_{k-1}\left(i\right)=f^{-1}\left[a+\delta q_{4,k-1}\left(i\right)\right]\chi_{k-1}^{\delta ℜ}\left(i\right)$
Compute the quaternion sigma points $\chi_{k-1}^{q}\left(i\right)=\chi_{k-1}^{\delta q}\left(i\right)⊗{\hat{q}}_{k-1}$, then $\chi_{k-1}^{}\left(i\right)=\left[\chi_{k-1}^{q}\left(i\right);\chi_{k-1}^{e}\left(i\right)\right]$
Propagate sigma points $\chi_{k/k-1}^{}\left(i\right)=f\left(\chi_{k-1}^{}\left(i\right)\right)=\left[\chi_{k/k-1}^{q}\left(i\right);\chi_{k/k-1}^{e}\left(i\right)\right]$
Compute error quaternion sigma points$\chi_{k/k-1}^{\delta q}\left(i\right)=\chi_{k/k-1}^{q}\left(i\right)⊗{\left[x_{k/k-1}^{\bar{q}}\right]}^{-1}$ and $\chi_{k/k-1}^{\delta q}\left(i\right)=\left[\delta \rho_{k/k-1}^{}\left(i\right);\delta q_{4,k/k-1}\left(i\right)\right]$
Compute GRP sigma points$\chi_{k/k-1}^{\delta ℜ}\left(i\right)=f\frac{\delta \rho_{k/k-1}\left(i\right)}{a+\delta q_{4,k/k-1}\left(i\right)}$ and $\chi_{k/k-1}^{}\left(i\right)=\left[\chi_{k/k-1}^{\delta ℜ}\left(i\right);\chi_{k/k-1}^{e}\left(i\right)\right]$
The predicted mean and covariance of state
${\hat{x}}_{k/k-1}=\sum_{i=0}^{2n}w\left(i\right)\chi_{k/k-1}\left(i\right)$

$P_{k/k-1}=\sum_{i=0}^{2n}w\left(i\right)\left(\chi_{k/k-1}\left(i\right)-{\hat{x}}_{k/k-1}\right){\left(\chi_{k/k-1}\left(i\right)-{\hat{x}}_{k/k-1}\right)}^{T}+W_{k-1}$
*Measurement update*:Compute filtering gain,
$K_{k}=P_{k/k-1}H_{k}^{T}{\left[H_{k}P_{k/k-1}H_{k}^{T}+R_{k}\right]}^{-1}$
Compute posterior mean and covariance, ${\hat{x}}_{k}={\hat{x}}_{k/k-1}^{}+K_{k}\left(y_{k}-H_{k}{\hat{x}}_{k/k-1}^{}\right)$ and ${\hat{x}}_{k}=\left[{\hat{x}}_{k-1}^{\delta ℜ};{\hat{x}}_{k}^{e}\right]$$P_{k}=(I_{n\times n}-K_{k}H_{k})P_{k/k-1}$ with $I_{n\times n}$ is an *n*-dimension unit matrix*Attitude update*:The ${\hat{x}}_{k}^{\delta ℜ}$ corresponding quaternion is
${\hat{x}}_{k}^{\delta q}\left(i\right)={\left[\delta \rho_{k}^{}\left(i\right),\delta q_{k,4}\left(i\right)\right]}^{T}$
with$\delta q_{4,k}^{}=\frac{-a{\left\|{\hat{x}}_{k}^{\delta ℜ}\right\|}^{2}+f\sqrt{f^{2}+\left(1-a^{2}\right){\left\|{\hat{x}}_{k}^{\delta ℜ}\right\|}^{2}}}{f^{2}+{\left\|{\hat{x}}_{k}^{\delta ℜ}\right\|}^{2}}$ and $\delta \rho_{k}^{}=f^{-1}\left[a+\delta q_{4,k}\right]{\hat{x}}_{k}^{\delta ℜ}$
The update of quaternion is given by ${\hat{x}}_{k}^{q}={\hat{x}}_{k}^{\delta q}⊗x_{k/k-1}^{\bar{q}}$ and $\delta {ℜ}_{k}\Rightarrow 0$, ${\hat{x}}_{k}^{}(q)=\left[{\hat{x}}_{k}^{q};{\hat{x}}_{k}^{e}\right]$
Then enter the next filtering cycle.

### 3.3. Three-Dimensional Integrated Navigation Filters

For 3D attitude representations, like MRP and Euler angle, their integrated navigation filters help avoid singularity. In this section, we briefly show this problem.

According to definition of the family of Rodrigues parameters:

(46) $$RP=e\tan(\frac{\vartheta }{2N})$$

where the MRP $\sigma =e\tan(\frac{\vartheta }{4})$ when N = 2, and ϑ is the attitude rotation vector. Thus, we know that the singularity appears at 2π±4nπ where *n* is a positive integer. Traditionally, the MRP can be transferred to SMRP $\sigma^{s}$ to avoid singularity. The relationship between MRP and SMRP is:

(47) $$\sigma^{s}=-\frac{\sigma }{{\left\|\sigma \right\|}^{2}}$$

Since the SMRP is defined by $\sigma^{s}=-e\cot(\frac{\vartheta }{4})$, the singularities of MRP and SMRP are different. Thus, the MRP-UKF exploits this feature to avoid singularity. For MRP-UKF, we not only consider the switch in attitude representation, but also the switch in filtering covariance. The switch in attitude representation affects the stability of filtering covariance. To stabilize the filtering covariance, the switch of filtering covariance in MRP-UKF can be shown by:

(48) $$A=\left(1-{\left(\sigma \right)}^{T}\sigma \right)I_{3\times 3}+2\left[\sigma \times \right]+2\sigma {\left(\sigma \right)}^{T}$$

(49) $$T=diag\left(\left[A;I_{n\times n}\right]\right)$$

(50) $$A^{s}=\left(1-{\left(\sigma^{s}\right)}^{T}\sigma^{s}\right)I_{3\times 3}+2\left[\sigma^{s}\times \right]+2\sigma^{s}{\left(\sigma^{s}\right)}^{T}$$

(51) $$T^{s}=diag\left(\left[A^{s};I_{n\times n}\right]\right)$$

(52) $$P^{s}=\frac{(T^{s}T^{T})P{\left(T^{s}T^{T}\right)}^{T}}{{\left(1+\sigma^{T}\sigma \right)}^{4}}$$

**Figure 3:**
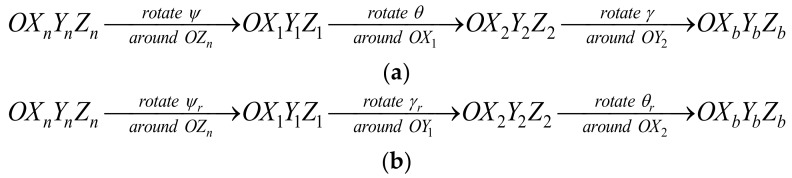
(**a**)The forward rotate order of Euler angle; (**b**) the inverted rotate order of Euler angle.

For the Euler angle in UKF compared with MRP, the singularity of the Euler angle is complex because the singularity is related to the rotate order of Euler angle for the attitude transformation matrix. Different rotate orders of the Euler angle in the attitude transformation matrix will have different singularities. The DoEuler-UKF exploits this feature to avoid singularity. DoEuler-UKF uses two different rotate orders of Euler angles to avoid singularity. One of the Euler angles is called forward Euler angle (Figure 3a) and the attitude transformation matrix is outlined by Equation (4) and the corresponding attitude kinematic equation is outlined in Equation (3).

The other Euler angle is called the inverted Euler angle (Figure 3b) and the attitude transformation matrix is given by:

(53) $$C_{n}^{b}(\varphi_{r})=\left[\begin{array}{ccc} \cos\gamma_{r}\cos\psi_{r} & \cos\gamma_{r}\sin\psi_{r} & -\sin\gamma_{r}\\ \cos\psi_{r}\sin\gamma_{r}\sin\theta_{r}-\cos\theta_{r}\sin\psi_{r} & \cos\psi_{r}\cos\theta_{r}+\sin\psi_{r}\sin\gamma_{r}\sin\theta_{r} & \cos\gamma_{r}\sin\theta_{r}\\ \sin\psi_{r}\sin\theta_{r}+\cos\psi_{r}\cos\theta_{r}\sin\gamma_{r} & \cos\theta_{r}\sin\psi_{r}\sin\gamma_{r}-\cos\psi_{r}\sin\theta_{r} & \cos\gamma_{r}\cos\theta_{r} \end{array}\right]$$

where $\varphi_{r}=\left[\theta_{r};\gamma_{r};\psi_{r}\right]$ is the inverted Euler angle. Then, the corresponding attitude kinematic equation is given by:

(54) $$\left[\begin{array}{c} {\dot{\theta }}_{r}\\ {\dot{\gamma }}_{r}\\ {\dot{\psi }}_{r} \end{array}\right]=\frac{1}{\cos\gamma_{r}}\left[\begin{array}{ccc} \cos\gamma_{r} & \sin\gamma_{r}\sin\theta_{r} & \sin\gamma_{r}\cos\theta_{r}\\ 0 & \cos\theta_{r}\cos\gamma_{r} & -\sin\theta_{r}\cos\gamma_{r}\\ 0 & \sin\gamma & \cos\theta_{r} \end{array}\right]\omega_{nb}^{b}$$

The singularity of the forward Euler angle occurs when pitch θ→±π/2 and the singularity of the inverted Euler angle occurs when roll $\gamma_{r}\to \pm \pi /2$. Thus, the DoEuler-UKF uses this different singularity to switch the attitude to avoid singularity. Compared with MRP-UKF, the DoEuler-UKF do not seem to need switching of the filtering covariance due to the lack of change in the attitude representation.

### 3.4. Discussionof Integrated Navigation Filters

To highlight the characteristics of each algorithm, some remarks are provided and summarized.

Firstly, the states of the Euler-KF are state errors. The linear model of the Euler-KF system model involves accuracy lossy equations. Therefore, the estimation accuracy is low. However, the calculation amount is small. The integrated structure of navigation devices is more flexible and easier to implement and may be used to form a multi model system.

Secondly, the states of the MEKF are navigation parameters. The system model of MEKF includes basic SINS equations, which are accuracy lossless equations. The research core of MEKF involves determining how to acquire the attitude error to propagate filtering covariance. This is mainly reflected in Equations (13)–(15). MEKF has the advantages of EKF, but also has the disadvantages of EKF. Although MEKF does not have model accuracy loss, filtering accuracy loss occurs. Because MEKF is also easy to implement, it is widely used in engineering.

Thirdly, the states of USQUE are navigation parameters. The system model of USQUE includes basic SINS equations, which are also accuracy lossless equations. UKF has higher accuracy compared to EKF. However, the quaternion cannot be directly applied to UKF. The USQUE is centered on the idea of layered filtering. The filtering process is divided into two layers: the inner layer filtering uses GRP to update the inner attitude state, and the outer layer filtering uses the quaternion to propagate the global attitude state. This attitude transformation is depicted in Figure 2. Compared with other algorithms, USQUE has a unique attitude update that transforms GRP to a quaternion.

Finally, MRP-UKF and DoEuler-UKF are 3D attitude integrated navigation filters. The states of those filters are navigation parameters. The system models are also basic SINS equations. Compared with MEKF and USQUE using a global nonsingular quaternion, MRP-UKF and DoEuler-UKF avoid the attitude singularity problem. Some other studies focused on how to realize the switching of filtering covariance or attain stable filtering.

## 4. Simulation Test and Experiments

A simulation test and a car-mounted experiment were conducted to comprehensively compare the performance of the five filtering methods. The car-mounted experiment used MEMS/GPS integration. From the analysis of the results, we determined the merits of the different integrated navigation filters under different conditions. Thus, we are able to provide some useful suggestions for the selection of integrated navigation filters.

### 4.1. Simulation

**Figure 4:**
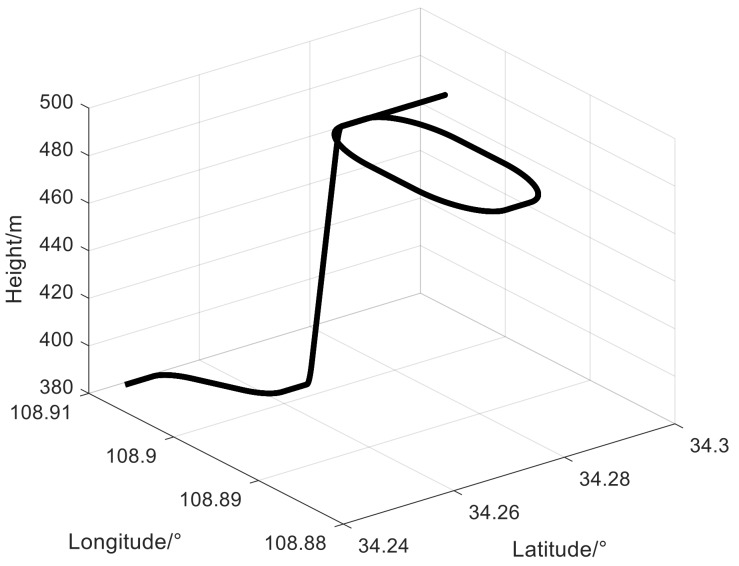
The simulation test trajectory.

We compared the estimation performance for attitude, position, velocity, and computing. We conducted a loosely coupled SINS/GPS test with velocity as the measurement. The simulation test trajectory will be shown in Figure 4. The total simulation time was *N* = 1300 s. The total Monte-Carlo time was *M* = 50. In this simulation test, two metrics based on the mean error (ME) were used for the filtering accuracy check: *ME1* is the performance of a single Monte-Carlo run and *ME2* is the performance of the whole Monte-Carlo runs expressed in simulation time.

ME1 is defined as:

$$\begin{array}{cc} ME1(m)={\left(\frac{1}{N}\sum_{k=1}^{N}{\left(x_{ref,k}-{\hat{x}}_{k}\right)}_{k}\right)}_{m} & \;m=1,2,\ldots ,M \end{array}$$

ME2 is defined as:

$$\begin{array}{cc} ME2(k)={\left(\frac{1}{M}\sum_{m=1}^{M}{\left(x_{ref,k}-{\hat{x}}_{k}\right)}_{m}\right)}_{k} & \;k=1,2,\ldots ,N \end{array}$$

where $x_{ref,k}$ is the reference state, ${\hat{x}}_{k}$ is the estimate, *k* is the number of simulation, and *m* is the number of Monte-Carlo runs. In this test, ME1 was used for the position and velocity estimates and ME2 was used for the attitude estimate. The initialization conditions are shown in Table 4.

The states are defined by 15-dimensional vector include attitude, velocity, position, and inertial device errors.

$$P_{0}=diag{\left(\left[P_{q_{0}};P_{v_{0}};P_{p_{0}};P_{\varepsilon_{0}};P_{\nabla_{0}}\right]\right)}^{2}\;\mathrm{with}\;P_{q_{0}}={\left[3.0462e^{-4},3.0462e^{-4},3.0462e^{-4}\right]}^{T}$$

$$P_{v_{0}}={\left[0.01,0.01,0.01\right]}^{T},\;P_{p_{0}}={\left[2.4582e^{-14},2.4582e^{-14},1\right]}^{T},$$

$$P_{\varepsilon_{0}}={\left[2.3504e^{-11},2.3504e^{-11},2.3504e^{-11}\right]}^{T},\;P_{\nabla_{0}}={\left[9.5655e^{-5},9.5655e^{-5},9.5655e^{-5}\right]}^{T}$$

$$Q_{0}=diag{\left(\left[0.01;0.001;100;10\right]\right)}^{2}$$

$$R_{0}=diag{\left(\left[R_{v_{0}}\right]\right)}^{2}R_{v_{0}}={\left[0.01,0.01,0.01\right]}^{T}$$

**Figure 5:**
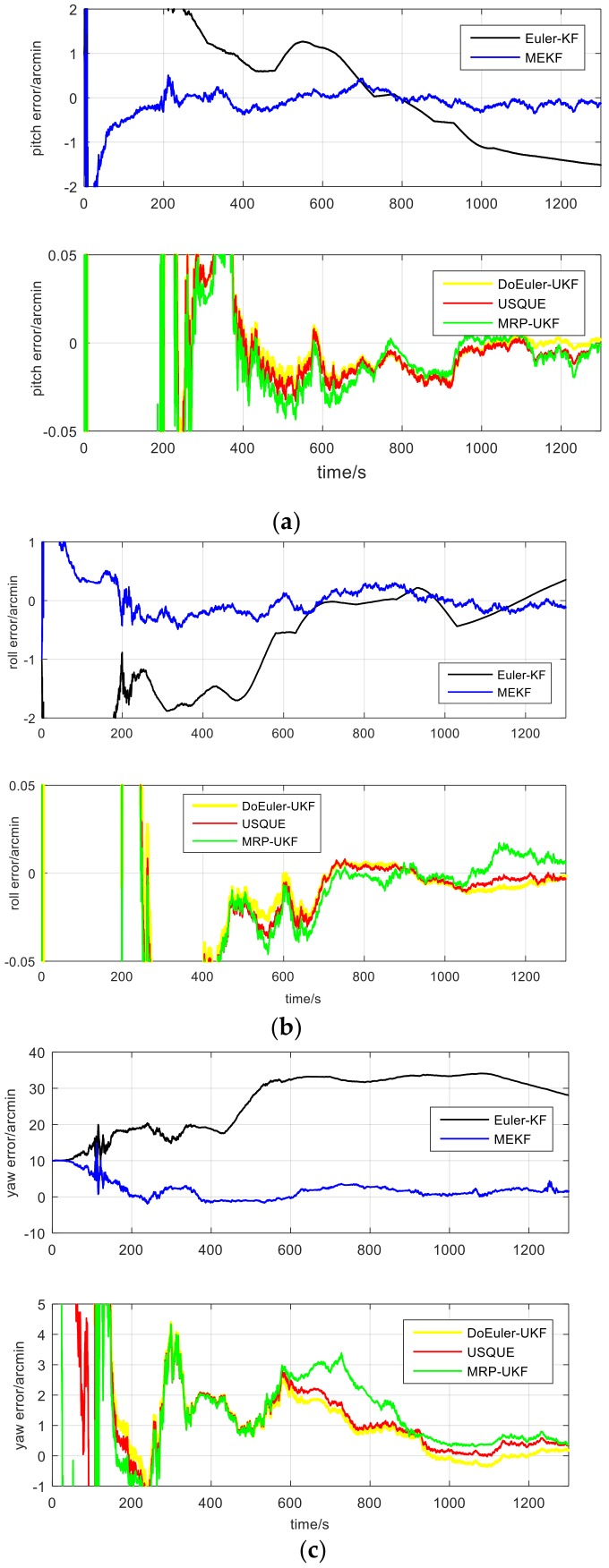
(**a**) Pitch errors of each filter in ME2; (**b**) Roll errors of each filter in ME2; (**c**) Yaw errors of each filter in ME2.

**Figure 6:**
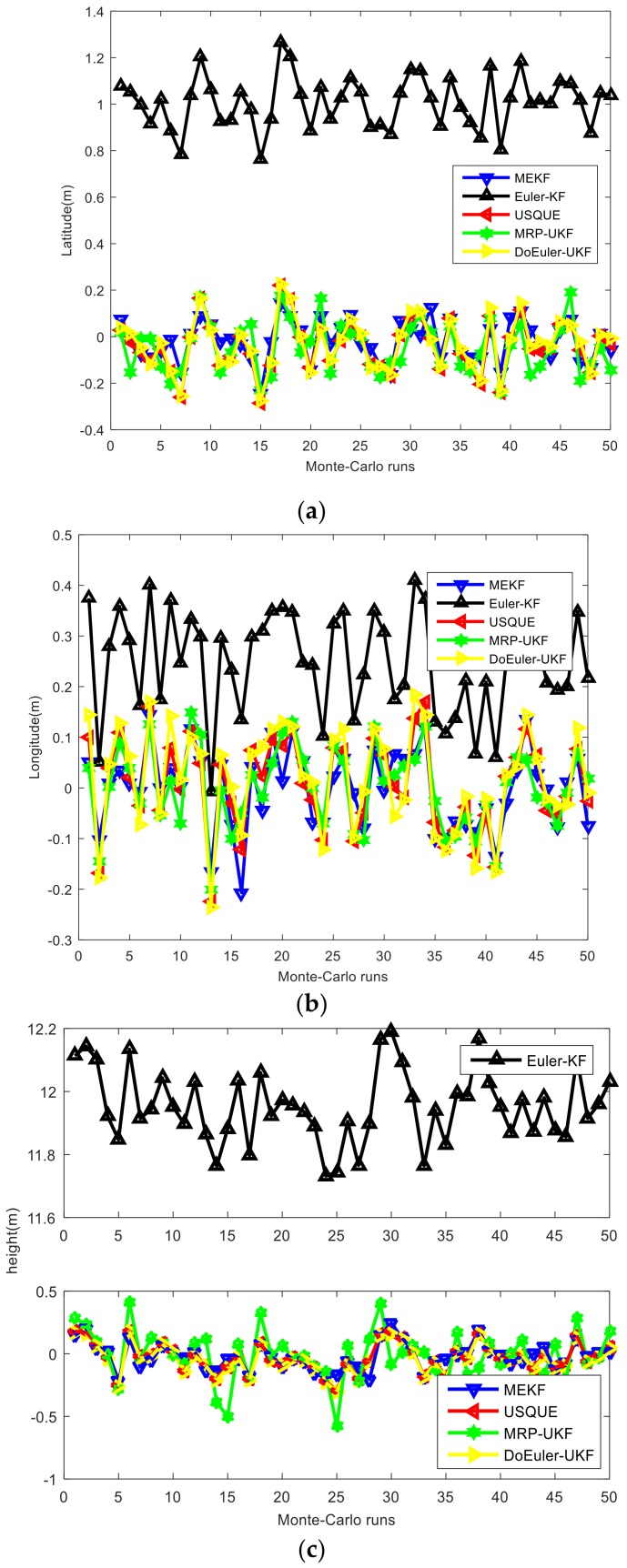
(**a**) Latitude errors of each filter; (**b**) Longitude errors of each filter; (**c**) Height errors of each filter.

**Figure 7:**
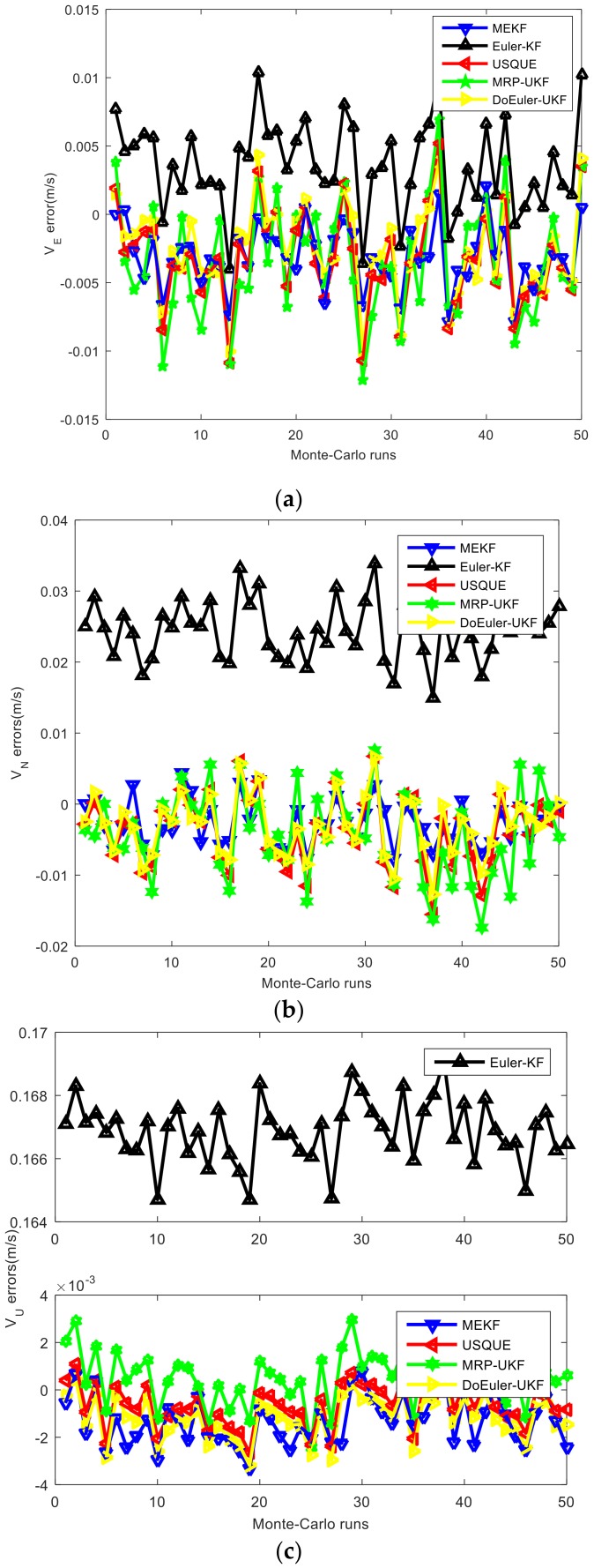
(**a**) East velocity errors of each filter; (**b**) North velocity errors of each filter; (**c**) Up velocity errors of each filter.

In the loosely-coupled strapdown inertial integrated navigation system with velocity as the measurement, the estimation performance for attitude, position, and velocity of the five methods are shown in Figure 5, Figure 6 and Figure 7 and Table 5. The average estimation errors equations in Table 5 are given by:

$$\delta \hat{x}=\frac{\sum_{i=0}^{T}\sum_{m=1}^{M}\left|{\hat{x}}_{i}^{m}\right|}{MT}\begin{array}{cc} & i=0,1,\ldots ,T\begin{array}{cc} & m=1,2,\ldots ,M \end{array} \end{array}$$

where $\delta \hat{x}$ is the average estimation error, M is the number of total Monte-Carlo simulations, T is the total simulation time, and $\left|{\hat{x}}_{i}^{m}\right|$ is the absolute value of the error.

According to the simulation results, regardless of attitude position and velocity, the estimation accuracies of UKF series filters are better than EKF series filters. The nonlinear model filter is more accurate than the linear model filter. USQUE, DoEuler-UKF, and MRP-UKF are almost equal in estimation accuracy, whereas Euler-KF is the worst. In attitude estimation, MEKF and USQUE are more stable than Euler-KF, DoEuler-UKF, and MRP-UKF. In terms of computation time, Euler-KF and MEKF have a considerable advantage; DoEuler-UKF is also far better than USQUE and MRP-UKF. When comparing MRP-UKF and USQUE, MRP-UKF is slightly better. The equations in USQUE, MEKF, MRP-UKF, and DoEuler-UKF are nonlinear basic SINS equations, whereas the Euler-KF equations are linear SINS error equations. To meet the linear model requirements, SINS error equations usually include partial ellipsis and linearization approximation, but SINS equations are more precise. As a result, USQUE, MEKF MRP-UKF, and DoEuler-UKF need more computation time. MEKF is a special form of EKF that is worse than UKF in estimation accuracy. Although MEKF is linearized, the Jacobian matrix of MEKF is created to propagate covariance and is unrelated to the propagation of the state. MEKF is worse than UKF series filters, but better than Euler-KF in terms of estimation accuracy. For UKF series filters researched in this paper, MRP-UKF, and DoEuler-UKF, due to the switching problem, the estimation accuracy is also slightly inadequate. As the model used in USQUE has the highest degree of nonlinearity, it has the longest computation time.

Fortightly-coupled or super tightly-coupled systems, the measurement model becomes nonlinear. USQUE MRP-UKF and DoEuler-UKF require more computation time and the estimation accuracy of Euler-KF worsens. MEKF is the first choice both in accuracy or computation time. Through many iterations, MEKF estimation accuracy is almost equal to USQUE and it needs less computation time. This explains why EKF series filters are widely used in engineering.

### 4.2. Car-Mounted Experiment

In the car-mounted experiments, MEMS was used for low-precision SINS to examine the performance of the five filters. The car-mounted experimental platform included MEMS strapdown inertial measurement equipment XW-IMU5220, a navigation-grade ring laser SINS for attitude reference, and a GPS receiver.

For the MEMS/GPS car-mounted experiment, we chose attitude, velocity, position, and gyro bias for comparison. In this car-mounted experiment, we focused on the estimated performance of attitude and gyro bias with the velocity and position measurements obtained from the GPS. The specifications of the gyroscopes and accelerometers of the MEMS IMU are listed in Table 6. The accuracy of the GPS receiver was 0.1 m/s for velocity and less than 2 m for position. Since the performance of high-precision laser SINS is much better than the low-precision MEMS, a highly-precise laser SINS was chosen for the attitude reference system. The specifications of MEMS and some initialization conditions are provided in Table 7.

$$P_{0}=diag{\left(\left[P_{q_{0}};P_{v_{0}};P_{p_{0}};P_{\varepsilon_{0}}\right]\right)}^{2}$$

$$\mathrm{with}\;P_{q_{0}}={\left[3.0462e^{-4},3.0462e^{-4},3.0462e^{-4}\right]}^{T},\;P_{v_{0}}={\left[0.01,0.01\right]}^{T},\;P_{p_{0}}={\left[2.4582e^{-14},2.4582e^{-14}\right]}^{T}$$

$$P_{\varepsilon_{0}}={\left[2.3504e^{-11},2.3504e^{-11},2.3504e^{-11}\right]}^{T}$$

$$Q_{0}=diag{\left(\left[0.01;0.001;100;10\right]\right)}^{2}$$

$$R_{0}=diag{\left(\left[R_{v_{0}};R_{p_{0}}\right]\right)}^{2}$$

$$R_{v_{0}}={\left[0.01,0.01\right]}^{T};\;R_{p_{0}}={\left[2.4582e^{-14},2.4582e^{-14}\right]}^{T}$$

The experiment test trajectory involved driving the car around on the ground. The experiment time was 250 s.

**Figure 8:**
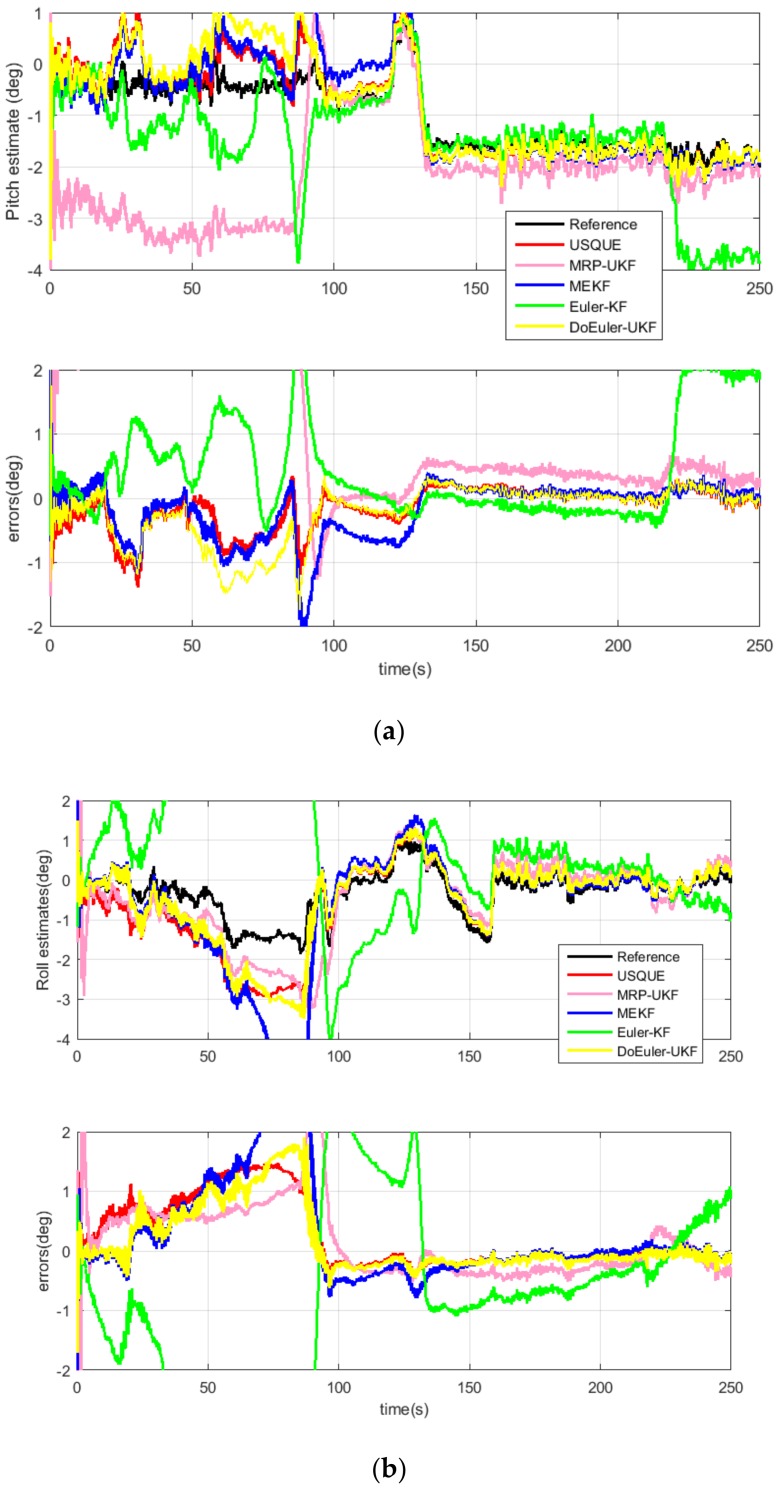

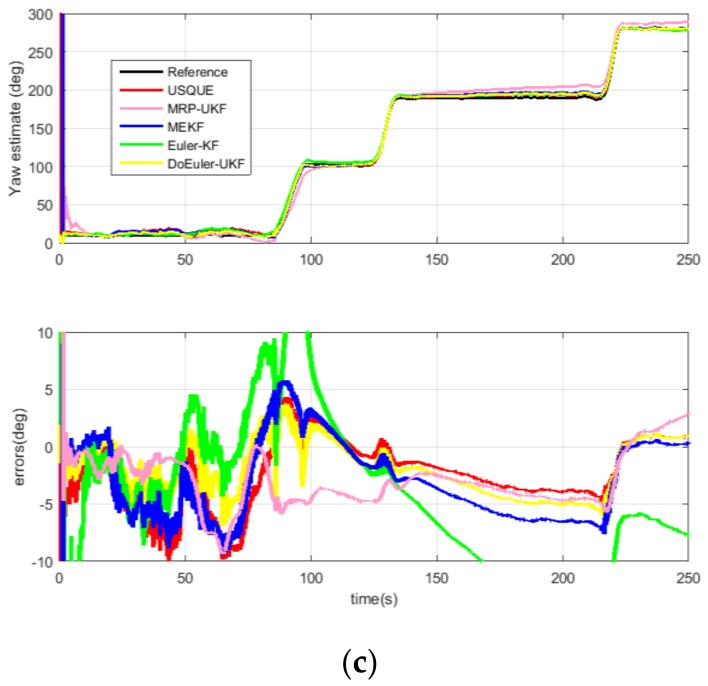
(**a**) Pitch estimate of each filter; (**b**) Roll estimate of each filter; (**c**) Yaw estimate of each filter.

**Figure 9:**
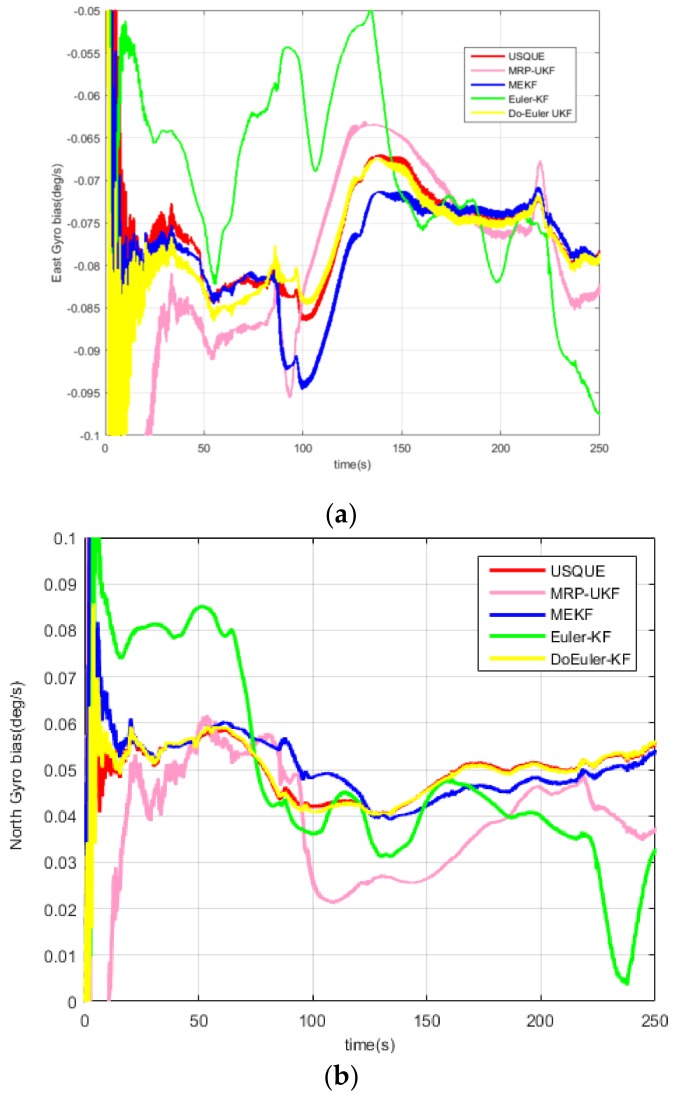

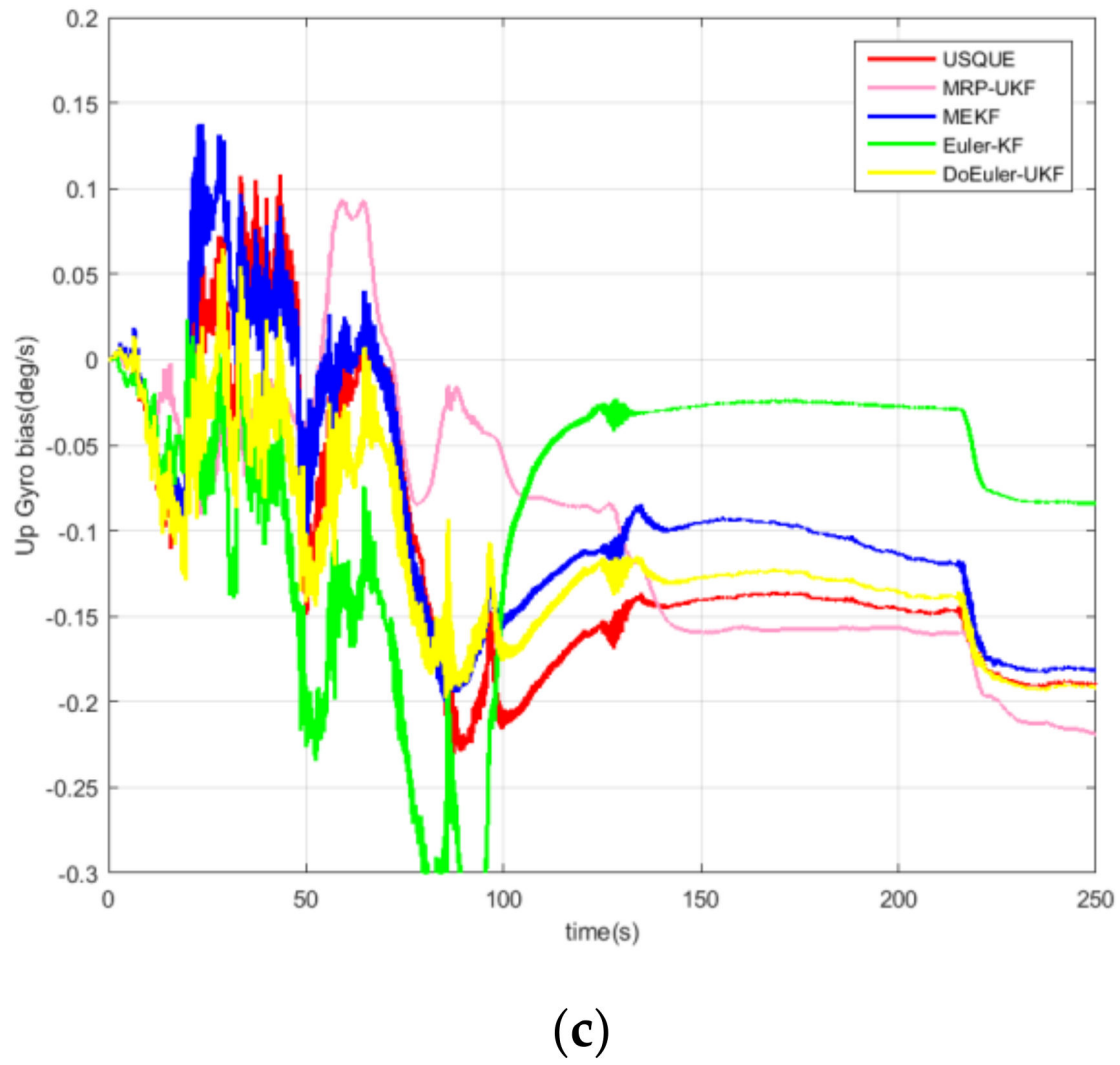
(**a**) East gyro bias estimate of each filter; (**b**) North gyro bias estimate of each filter; (**c**) Up gyro bias estimate of each filter.

For the MEMS-based SINS, the estimation performance for attitude and gyro bias of the five filters are displayed in Figure 8 and Figure 9 and Table 8. The average estimation errors equation in Table 8 are given by:

$$\delta \hat{x}=\frac{\sum_{i=0}^{T}\left|{\hat{x}}_{i}^{}\right|}{T}\begin{array}{cc} & i=0,1,\ldots ,T \end{array}$$

where $\delta \hat{x}$ is the average estimation error, T is the total experience time, and $\left|{\hat{x}}_{i}\right|$ is the absolute value of the error.

According to the experiment, both in attitude and gyro bias, it is found that the estimation performance of USQUE is better than MEKF. This is because the MEKF algorithm uses the same model as USQUE. However, in the attitude estimation part, in order to calculate the Jacobian matrix, the δα approximation is used instead of δq, with some precision loss. In addition, the use of the Jacobian matrix also has loss of precision, and Euler-KF uses the SINS error equation. The model makes some approximations in the linearization process, so that although the Kalman filter is optimal in the linear model estimation, because of the model’s precision loss, the estimation accuracy is the worst. The accuracy of the model and calculation method is a double-edged sword. The high precision comes at the cost of calculation. DoEuler-UKF has a high estimation performance both in accuracy and computation time. When comparing with USQUE, the accuracy is almost equal and the computation time is half, because of the Euler-angle having less computation than quaternion. When using the MRP-UKF estimate of pitch and roll, the accuracy is pretty good, but when estimating gyro bias, the accuracy of MRP-UKF is a little poor.

According to the results and analysis of the above integrated navigation experiments, we drew some conclusions about the various indicators of the five filters. Table 9 summarizes the five filtering algorithm properties, characteristics, and estimation performances.

Table 9 shows that Euler-KF requires the least amount of calculation, but the estimation accuracy is the worst due to its linear model. Euler-KF is a mature integrated filtering method. The amount of calculation of MEKF is fairly small and the estimation accuracy is moderate. Although MEKF does not lose model accuracy, it loses filtering accuracy. MEKF is widely used in engineering. USQUE requires a huge amount of calculation, but has the best estimation accuracy. At present, USQUE is a research hotspot. MRP-UKF requires a large amount of calculation and moderate estimation accuracy. Notably, DoEuler-UKF requires a moderate amount of calculation, but its estimation accuracy is almost the same as USQUE. DoEuler-UKF produces good integrated navigation filtering performance. However, scholars have not paid enough attention to DoEuler-UKF.

## 5. Conclusions

We provide the following principles and recommendations for the use of integrated navigation filtering methods according to our research on the five filters.

First, USQUE has high estimation accuracy and high computational cost. For situations with high-accuracy estimate requirements, especially for aeronautics and astronautics, the calculation cost is secondary because high accuracy is required to meet the safety and stability requirements. Thus, it is generally recommended to use USQUE. MEKF has slightly lower accuracy than USQUE with a reasonable calculation cost. For nonlinear measurement functions, MEKF has higher accuracy through iterations with minimal calculation cost. Thus, MEKF is widely used for practical engineering. DoEuler-UKF has almost the same accuracy as USQUE, while having a moderate calculation cost, but it is not as stable as USQUE or MEKF. In some relatively stable systems, DoEuler-UKF will perform the best. MRP-UKF has moderate accuracy and high computational cost. Finally, in situations with slow motion and relatively low accuracy requirements, Euler-KF would be suitable for application because the filtering function of Euler-KF is linear and easy to implement and understand.

## Figures and Tables

**Table 1:** Calculation equations of Jacobian matrix.

$F_{11}=-\left[({\tilde{\omega }}_{ib}^{b}-\hat{\varepsilon })\times \right]$	$F_{12}=-C_{n}^{b}(\hat{q}){\left.\frac{\partial \omega_{in}^{n}}{\partial p}\right|}_{\hat{p},\hat{v}}$	$F_{13}=-C_{n}^{b}(\hat{q}){\left.\frac{\partial \omega_{in}^{n}}{\partial v}\right|}_{\hat{p}}$	$F_{14}=I_{3\times 3}$	$0_{3\times 3}$
$0_{3\times 3}$	$F_{22}={\left.\frac{\partial \dot{p}}{\partial p}\right|}_{\hat{p},\hat{v}}$	$F_{23}={\left.\frac{\partial \dot{p}}{\partial v}\right|}_{\hat{p}}$	$0_{3\times 3}$	$0_{3\times 3}$
$F_{31}=-C_{b}^{n}(\hat{q})\left[f^{b}\times \right]$	$F_{32}={\left.\frac{\partial \dot{v}}{\partial p}\right|}_{\hat{p},\hat{v}}$	$F_{33}={\left.\frac{\partial \dot{v}}{\partial v}\right|}_{\hat{p},\hat{v}}$	$0_{3\times 3}$	$F_{35}=-C_{b}^{n}(\hat{q})$

**Table 2:** Calculation equations of velocity partial differentials.

$Y_{11}=\frac{v_{E}v_{N}\sec^{2}\lambda }{R_{N}+h}-\frac{v_{E}v_{N}\tan\lambda }{{\left(R_{N}+h\right)}^{2}}\frac{\partial R_{N}}{\partial \lambda }+2\omega_{ie}^{e}v_{N}\cos\lambda +\frac{v_{E}v_{U}}{{\left(R_{N}+h\right)}^{2}}\frac{\partial R_{N}}{\partial \lambda }+2\omega_{ie}^{e}v_{U}\sin\lambda$
$Y_{13}=\frac{v_{E}v_{U}-v_{E}v_{N}\tan\lambda }{{\left(R_{N}+h\right)}^{2}}$
$Y_{21}=-\frac{v_{E}^{2}\sec^{2}\lambda }{R_{N}+h}+\frac{v_{E}^{2}\tan\lambda }{{\left(R_{N}+h\right)}^{2}}\frac{\partial R_{N}}{\partial \lambda }-2\omega_{ie}^{e}v_{E}\cos\lambda +\frac{v_{E}v_{U}}{{\left(R_{M}+h\right)}^{2}}\frac{\partial R_{M}}{\partial \lambda }$
$Y_{23}=\frac{v_{E}^{2}\tan\lambda }{{\left(R_{N}^{}+h\right)}^{2}}+\frac{v_{N}v_{U}}{{\left(R_{M}^{}+h\right)}^{2}}$
$Y_{31}=-\frac{v_{E}^{2}}{{\left(R_{N}^{}+h\right)}^{2}}\frac{\partial R_{N}}{\partial \lambda }-\frac{v_{N}^{2}}{{\left(R_{M}^{}+h\right)}^{2}}\frac{\partial R_{M}}{\partial \lambda }-2\omega_{ie}^{e}v_{E}\sin\lambda -\frac{\partial g}{\partial \lambda }$
$Y_{33}=-\frac{v_{E}^{2}}{{\left(R_{N}^{}+h\right)}^{2}}-\frac{v_{N}^{2}}{{\left(R_{M}^{}+h\right)}^{2}}-\frac{\partial g}{\partial h}$

**Table 3:** Calculation equations of velocity partial differentials.

$Z_{11}=\frac{-v_{U}+v_{N}\tan\lambda }{R_{N}+h}$	$Z_{12}=\frac{v_{E}\tan\lambda }{R_{N}+h}+2\omega_{ie}^{e}\sin\lambda$	$Z_{13}=-\frac{v_{E}}{R_{N}+h}-2\omega_{ie}^{e}\cos\lambda$
$Z_{21}=\frac{-2v_{E}\tan\lambda }{R_{N}+h}+2\omega_{ie}^{}\sin\lambda$	$Z_{22}=\frac{-v_{U}}{R_{M}+h}$	$Z_{23}=\frac{-v_{N}}{R_{M}+h}$
$Z_{31}=\frac{2v_{E}}{R_{N}+h}+2\omega_{ie}^{e}\cos\lambda$	$Z_{32}=\frac{2v_{N}}{R_{M}+h}$	0

**Table 4:** Initialization conditions.

Filter	Initialization State	Filtering Covariance	System Noise Covariance	Measurement Noise Covariance
USQUE/MEKF	${\hat{x}}_{0}(q_{0})$	$P_{0}$	$Q_{0}$	$R_{0}$
MRP-UKF	${\hat{x}}_{0}(\sigma_{0})$, $\sigma_{0}=\rho /\left(1+q_{4}\right)$			
DoEuler-UKF	${\hat{x}}_{0}(\varphi_{0})$, $\varphi_{0}\leftarrow q_{0}$			
Euler-KF	${\hat{x}}_{0}(0)$			

**Table 5:** Average estimation error and computation time of each filter.

Average Estimation Error	Euler-KF	MEKF	USQUE	MRP-UKF	DoEuler-UKF
Pitch error (arcmin)	1.231	0.519	0.278	0.491	0.354
Roll error (arcmin)	1.069	0.529	0.378	0.564	0.457
Yaw error (arcmin)	24.064	18.563	9.625	11.688	9.969
Latitude error (m)	0.982	0.156	0.098	0.143	0.132
Longitude error (m)	0.245	0.177	0.154	0.167	0.162
Height error (m)	11.167	0.475	0.398	0.401	0.399
East velocity error (m/s)	0.00462	0.00652	0.00518	0.00531	0.00523
North velocity error (m/s)	0.0254	0.0072	0.0058	0.0092	0.0123
Up velocity error (m/s)	0.167	0.012	0.008	0.008	0.009
Computation time (s)	3.49	10.26	33.65	26.74	18.65

**Table 6:** Specifications of the XW-IMU5220.

Technical Indexes	Gyroscope	Accelerometer
Dynamic range	$\pm 150^{\;{}^{\circ}}/s$	±10 g
Bias	$\leq 0.5^{\;{}^{\circ}}/s$	≤0.005 g
Bias stability	$\leq 0.02^{\;{}^{\circ}}/s$	≤0.001 g
Bias repeatability	$\leq 0.02^{\;{}^{\circ}}/s$	≤0.002 g
Update rate	100 Hz	100 Hz

**Table 7:** The initialization conditions.

Filter	State	Initialization
MEKF	$q,v_{N},v_{E},\lambda ,\phi ,\varepsilon$	$q_{0}$
		$v_{N},v_{E}$ and λ,ϕ provided by laser SINS
		$\varepsilon =0_{3\times 1}$
USQUE	$q,v_{N},v_{E},\lambda ,\phi ,\varepsilon$	$q_{0}$ with $x^{\delta ℜ}=0_{3\times 1}$
		$v_{N},v_{E}$ and λ,ϕ provided by laser SINS
		$\varepsilon =0_{3\times 1}$
MRP-UKF	$\sigma ,v_{N},v_{E},\lambda ,\phi ,\varepsilon$	$\sigma_{0}$
		$v_{N},v_{E}$ and λ,ϕ provided by laser SINS
		$\varepsilon =0_{3\times 1}$
DoEuler-UKF	$\theta ,\gamma ,\psi ,\theta_{r},\gamma_{r},\psi_{r},v_{N},v_{E},\lambda ,\phi ,\varepsilon$	$\theta_{0},\gamma_{0},\psi_{0}/\theta_{r}{}_{0},\gamma_{r}{}_{0},\psi_{r}{}_{0}$
		$v_{N},v_{E}$ and λ,ϕ provided by laser SINS
		$\varepsilon =0_{3\times 1}$
Euler-KF	$\varphi ,\delta v_{N},\delta v_{E},\delta \lambda ,\delta \phi ,\delta \varepsilon$	$0_{10\times 1}$

**Table 8:** Average estimation error and computation time of each filter.

Average Estimation Error	Euler-KF	MEKF	USQUE	MRP-UKF	Do-Euler UKF
Pitch error (°)	4.871	0.321	0.239	1.179	0.284
Roll error (°)	1.843	0.592	0.215	0.485	0.424
Yaw error (°)	7.425	5.696	2.214	3.359	4.218
East gyro bias error (°/s)	0.0012	0.0014	0.0013	0.0014	0.0012
North gyro bias error (°/s)	8.69 × 10^−4^	8.82 × 10^−4^	7.36 × 10^−4^	8.66 × 10^−4^	8.51 × 10^−4^
Up gyro bias error (°/s)	0.0019	0.0022	0.0018	0.0014	0.0024
Computation time (s)	5.507	19.859	61.355	55.219	32.687

**Table 9:** Indicators of integrated navigation filters.

Integrated Filtering Method	Core Equation of Filtering Method	Amount of Calculation	Estimation Accuracy	Characteristic
Euler-KF	LinearEquation (10)	Least	Worst	Mature
MEKF	Nonlinear Table 1	Fairly small	Moderate	Widely used in engineering
USQUE	Nonlinear Table 2	Huge	Best	Research hotspots
MRP-UKF	Nonlinear Equations (29)–(31)	large	Moderate	Need improvement
DoEuler-UKF	Nonlinear Equations (32)–(33)	Moderate	Better	Potential

## References

[1] Alandry B., Latorre L., Mailly F., Nouet P. (2011). A fully integrated inertial measurement unit: Application to attitude and heading determination. IEEE Sens. J..

[2] Zhang L., Chen M., He H. A method research on robust fault diagnosis of integrated navigation systems. Proceedings of the 8th International Conference on Electronic Measurement and Instruments (IEEE).

[3] Li K., Chang L., Hu B. (2015). Unscented attitude estimator based on dual attitude representations. IEEE Trans. Instrum. Meas..

[4] Wu Y.X., Pan X.F. (2013). Velocity/position integration formula, Part I: Application to in-flight coarse alignment. IEEE Trans. Aerosp. Electron. Syst..

[5] Wang D., Xu X., Zhu Y. (2018). A Novel Hybrid of a Fading Filter and an Extreme Learning Machine for GPS/INS during GPS Outages. Sensors.

[6] Jalving B., Gade K., Svartveit K., Willumsen A.B., Sorhagen R. (2004). DVL Velocity Aiding in the HUGIN 1000 Integrated Navigation System.

[7] Qin Y., Zhang H. (2015). Kalman Filter and Integrated Navigation.

[8] Gu D.Q., El-Sheimy N., Hassan T., Syed Z. Coarse Alignment for Marine SINS Using Gravity in the Inertial Frame as a Reference. Proceedings of the IEEE/ION Position, Location and Navigation Symposium (PLANS).

[9] Ren H., Kazanzides P. (2012). Investigation of attitude tracking using an integrated inertial and magnetic navigation system for hand-held surgical instruments. IEEE/ASME Trans. Mechatron..

[10] Levinson E., Horst J. The next generation marine inertial navigator is here now. Proceedings of the IEEE Position Location and Navigation Symposium.

[11] Qin Y. (2005). Inertial Navigation.

[12] Chen Y. (2007). Principle of Inertial Navigation.

[13] Xu R., Ding M., Qi Y., Yue S. (2018). Performance Analysis of GNSS/INS Loosely Coupled Integration Systems under Spoofing Attacks. Sensors.

[14] Crassidis J.L., Markley F., Cheng Y. (2007). Survey of Nonlinear Attitude Estimation Methods. J. Guid. Control Dyn..

[15] Dissanayake G., Sukkarieh S., Nebot E. (2001). The aiding of a low-cost strapdown inertial measurement unit using vehicle model constraints for land vehicle applications. IEEE Trans. Robot. Autom..

[16] Sun F., Xia J.Z., Lan H.Y., Zhang Y. (2014). DVL-aided Paralled Algorithm for Marine Attitude and Heading Reference System. J. Comput. Inf. Syst..

[17] Liu Y., Liu F., Gao Y., Zhao L. (2018). Implementation and Analysis of Tightly Coupled Global Navigation Satellite System Precise Point Position/Inertial Navigation System with Insufficient Satellites for Land Vehicle Navigation. Sensors.

[18] Chang L., Li Y., Xue B. (2016). Initial Alignment for Doppler Velocity Log aided Strapdown Inertial Navigation System with Limited Information. IEEE/ASME Trans. Mechatron..

[19] Groves P.D. (2008). Principles of GNSS, Inertial, and Multisensor Integrated Navigation Systems.

[20] Wiener N. (1949). Extrapolation, Interpolation, and Smoothing of Stationary Time Series.

[21] Farrell J., Barth M. (1998). The Global Positioning System and Inertial Navigation.

[22] Wang Y., Sun S., Li L. (2014). Adaptively robust unscented Kalman filter for tracking a maneuvering vehicle. J. Guid. Control Dyn..

[23] Feng K., Li J., Zhang X., Zhang X., Shen C., Cao H. (2018). An Improved Strong Tracking Cubature Kalman Filter for GPS/INS Integrated Navigation Systems. Sensors.

[24] Zhou J., Knedlik S., Loffeld O. (2010). INS/GPS Tightly-coupled Integration using Adaptive Unscented Particle Filter. J. Navig..

[25] Jiao Z., Zhang R. (2013). Kalman/Particle Filter for Integrated Navigation System. Adv. Mater. Res..

[26] Hao Y., Xu A. (2018). A Modified Extended Kalman Filter for a Two-Antenna GPS/INS Vehicular Navigation System. Sensors.

[27] Costanzi R., Fanelli F., Monni N., Ridolfi A., Allotta B. (2016). An attitude estimation algorithm for mobile robots under unknown magnetic disturbances. IEEE/ASME Trans. Mechatron..

[28] Zhang L.J. (2013). Multiplicative filtering for spacecraft attitude determination. J. Natl. Univ. Def. Technol..

[29] Jia R. (2014). Attitude Estimation Algorithm for Low Cost MEMS Based on Quaternion EKF. Chin. J. Sens. Actuators.

[30] Qin F., Chang L., Jiang S., Zha F. (2018). A Sequential Multiplicative Extended Kalman Filter for Attitude Estimation Using Vector Observations. Sensors.

[31] Crassidis J.L., Markley F. (2003). Unscented Filtering for Spacecraft Attitude Estimation. J. Guid. Control Dyn..

[32] Crassidis J.L. (2006). Sigma-point Kalman filtering for integrated GPS and inertial navigation. IEEE Trans. Aerosp. Electron. Syst..

[33] Zhou J., Edwan E., Kinedlik S., Loffeld O. Low-cost INS/GPS with nonlinear filtering methods. Proceedings of the 13th IEEE Conference on Information Fusion.

[34] Li K., Hu B., Chang L. (2015). Modified Quaternion Unscented Kalman filter based on Modified Rodriguez Parameters. J. Navig..

[35] Karlgaard C.D., Schaub H. (2010). Nonsingluar attitude filtering using modified Rodrigues parameters. J. Astronaut. Sci..

[36] Ran C., Cheng X. (2016). A Direct and Non-singular UKF Approach using Euler angle kinematics for integrated navigation system. Sensors.

[37] Markley F.L., Crassidis J.L. (2014). Fundamentals of Spacecraft Attitude Determination and Control.

